# Kinectin 1 promotes the growth of triple-negative breast cancer via directly co-activating NF-kappaB/p65 and enhancing its transcriptional activity

**DOI:** 10.1038/s41392-021-00652-x

**Published:** 2021-07-05

**Authors:** Lin Gao, Shanze Chen, Malin Hong, Wenbin Zhou, Bilan Wang, Junying Qiu, Jinquan Xia, Pan Zhao, Li Fu, Jigang Wang, Yong Dai, Ni Xie, Qinhe Yang, Hsien-Da Huang, Xiang Gao, Chang Zou

**Affiliations:** 1 Department of Clinical Medical Research Center, The Second Clinical Medical College, Jinan University (Shenzhen People’s Hospital), The First Affiliated Hospital of Southern University of Science and Technology, Shenzhen, Guangdong PR China; 2 Integrated Chinese and Western Medicine Postdoctoral Research Station, Jinan University, Guangzhou, Guangdong PR China; 3 Department of Respiratory and Critical Care Medicine, First Affiliated Hospital of Southern University of Science and Technology; The Second Clinical Medical College, Jinan University (Shenzhen People’s Hospital), Shenzhen Institute of Respiratory Diseases, Shenzhen, Guangdong PR China; 4 Shenzhen Public Service Platform on Tumor Precision Medicine and Molecular Diagnosis, the Second Clinical Medical College, Jinan University (Shenzhen People’s Hospital), Shenzhen, Guangdong PR China; 5 Department of Pharmacy, West China Second University Hospital of Sichuan University, Chengdu, PR China; 6 Guangdong Provincial Key Laboratory of Regional Immunity and Diseases, Department of Pharmacology and International Cancer Center, Shenzhen University Health Science Center, Shenzhen, Guangdong China; 7 Department of Integrated Chinese and Western Medicine, Jinan University, Guangzhou, Guangdong PR China; 8 School of Life and Health Sciences, The Chinese University of Kong Hong, Shenzhen, China; 9 Warshel Institute for Computational Biology, The Chinese University of HongKong, Shenzhen, China; 10 Department of Neurosurgery, State Key Laboratory of Biotherapy, West China Hospital, Sichuan University, Chengdu, PR China

**Keywords:** Metastasis, Oncogenes

## Abstract

Triple-negative breast cancer (TNBC) is the most challenging subtype of breast cancer. Various endeavor has been made to explore the molecular biology basis of TNBC. Herein, we reported a novel function of factor Kinectin 1 (KTN1) as a carcinogenic promoter in TNBC. KTN1 expression in TNBC was increased compared with adjacent tissues or luminal or Her2 subtypes of breast cancer, and TNBC patients with high KTN1 expression have poor prognosis. In functional studies, knockdown of KTN1 inhibited the proliferation and invasiveness of TNBC both in vitro and in vivo, while overexpression of KTN1 promoted cancer cell proliferation and invasiveness. RNA-seq analysis revealed that the interaction of cytokine-cytokine receptor, particularly CXCL8 gene, was upregulated by KTN1, which was supported by the further experiments. CXCL8 depletion inhibited the tumorigenesis and progression of TNBC. Additionally, rescue experiments validated that KTN1-mediated cell growth acceleration in TNBC was dependent on CXCL8 both in vitro and in vivo. Furthermore, it was found that KTN1 enhanced the phosphorylation of NF-κB/p65 protein at Ser536 site, and specifically bound to NF-κB/p65 protein in the nucleus and cytoplasm of cells. Moreover, the transcription of CXCL8 gene was directly upregulated by the complex of KTN1 and NF-κB/p65 protein. Taken together, our results elucidated a novel mechanism of KTN1 gene in TNBC tumorigenesis and progression. KTN1 may be a potential molecular target for the development of TNBC treatment.

## Introduction

Triple-negative breast cancer (TNBC) is a, breast cancer (BCa) subtype with no expression of estrogen receptor (ER), progesterone receptor (PR), and human epidermal growth factor receptor 2 (Her2) protein. TNBC exhibits the highest recurrence and metastasis rate among all subtypes of BCa.^1^ Although TNBC accounts for only around 15% of all BCa, it remains the most intractable subtype of BCa.^2^ The challenge of TNBC treatment mainly attributes to its highly cell invasive capability and absence of effective biomarker for both early diagnosis and timely treatment. Therefore, more efforts need to be made to explore the molecular basis of cell invasion and disease progression in TNBC.

Kinectin 1 (KTN1), a multifunctional protein that interacts with Kinesin, participates in many processes of cellular dynamics such as the organelle motility and focal adhesion growth of cellular lamella.^3,4^ The interaction of KTN1 and kinesin is one of the important aspects of the cytoskeleton, which is essential for maintaining cell shape and cell migration. The molecular role of KTN1 in the modulation of gene transcription remains unknown in tumors. Recently studies indicated that overexpression of KTN1 in cutaneous squamous cell carcinoma and inhibition of KTN1 in these tumor cells can suppresses their cell proliferation via reducing the protein expression of epidermal growth factor receptor (EGFR).^5^ These findings implicate that KTN1 might associate with tumor progression. However, the molecular mechanism by which KTN1 promotes cancer cell growth remains largely unexplored.

Inflammatory response plays a key role in breast cancer development.^6^ The Nuclear factor kappaB (NF-κB) complex, which consists of five subunits, p50, p52, RelA (NF-κB/p65), RelB and c-Rel, plays a pivotal role in regulating inflammatory reaction. Interestingly, the NF-κB signaling pathway is activated in TNBC.^7^ In the canonical NF-κB pathway, the IκB kinase β (IKKβ)-catalyzed phosphorylating IκBα was stimulated by TNFα, IL-1β or IL-6. Degradation of IκBα protein complex led to the nuclear translocation of p50-p65 proteins.^8^ The majority of TNBC was composed of the basal-like and claudin-low BCa tissues. Previous studies showed that basal-like BCa tumors originate from the luminal progenitor cells,^7^ leading to increasing NF-κB activation levels compared to basal myoepithelial cells. However, the molecular mechanisms of NF-κB signaling pathway in regulating TNBC tumorigenesis is largely unclear. Mizuki et al. found that JAG1 plays a specific role in basal-like subtype cancer stem cells (CSCs) population of TNBC and is regulated by NF-κB signaling pathway, suggesting that NF-κB-JAG1 axis are primarily responsible for tumorigenesis and metastasis of TNBC.^7^

It is known that chronic irritation and inflammation may result in cancers. Activation of NF-κB is rapidly induced by external stimulus including pro-inflammatory cytokines.^9^ Cytokines and chemokines including Interleukin (IL)-6 and CXCLs are found to contribute to BCa growth, infiltration and,, chemotherapy resistance, and are involved in NF-κB signaling pathway.^10^ It is shown that the expression of intra-tumoral IL-1β protein is up-regulated in BCa compared to the adjacent tissues, indicating the oncogenic potential caused by the competitive binding of Interleukin-1 receptor type 2 (IL-1R2) to IL-1β.^11^ High IL-1R2 promotes the self-renewal and aggressive growth of breast tumor initiating cells.^12^ In addition, C-X-C motif chemokine ligand 8 [CXCL8; also named as interleukin-8 (IL-8)] participates in regulating cellular proliferation, migration, and invasiveness of cancer cells.^13^ Previous studies have demonstrated that high level of CXCL8 expression accelerated tumor cell proliferation, angiogenesis, and metastasis in gastrointestinal cancers.^14,15^ In BCa, activation of inflammatory cytokines by both autocrine and paracrine mechanisms has been found to contribute to elicit its aggressive growth, stemness, and chemoresistance.^16^ However, the molecular mechanism of upstream events involved in releasing these cytokines is ambiguous. Previous studies suggested that NF-κB, Jak/Stats and interferons might control these inflammatory cytokines in TNBC.^7,17^

In this study, we found that KTN1 was significantly upregulated in both TNBC tumor specimens and cells, and this upregulation positively associated with poor prognosis of TNBC patients. Both gene gain-of-function and loss-of-function analysis identified that KTN1 could promote TNBC cells growth and invasion both in vitro and in vivo. Moreover, KTN1 can enhance the gene transcription of proinflammatory cytokine CXCL8 and upregulate its expression both in mRNA and protein levels. Importantly, we found two potential nuclear localization signals (NLSs) of KTN1 protein domains, we found that KTN1 protein could interact with NF-κB/p65 in nuclei and increase its phosphorylation, which can further activate the transcriptional activities of NF-κB/p65 on CXCL8 gene promoter in TNBC. Furthermore, rescue experiments validated that KTN1-mediated promotion of cells growth in TNBC was dependent on CXCL8. Collectively, our findings highlight a brand-new transcription regulatory function of KTN1 in promoting TNBC cell growth, which could shed some lights on developing novel strategy for TNBC treatment.

## Results

### KTN1 is overexpressed in TNBC tumor

**Fig. 1:**
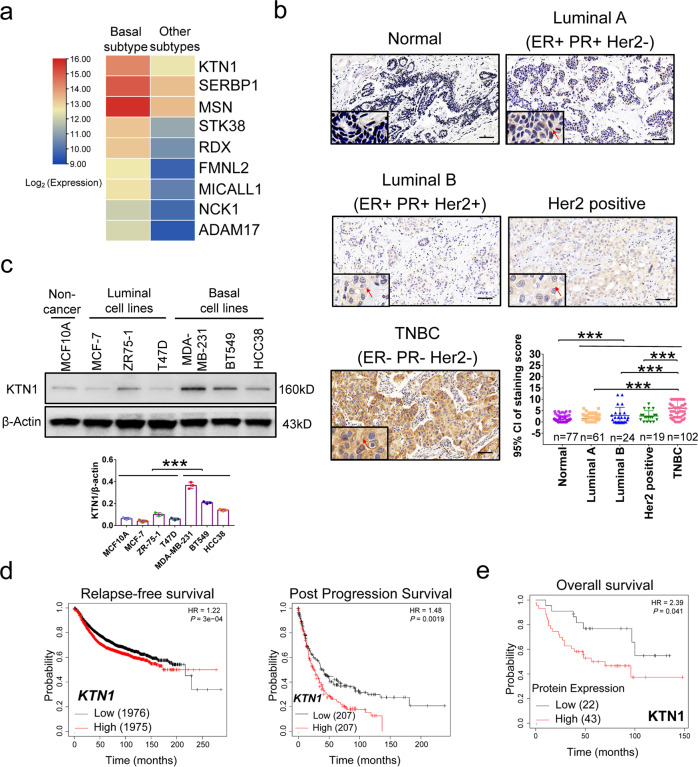
High KTN1 expression associated with clinical outcomes in TNBC tissues and cells. **a** Representative up-regulated genes in GO pathways. **b** Immunohistochemistry (IHC) staining analysis of KTN1 protein in different subtypes of BCa. Normal, *n* = 77; Luminal A, *n* = 61; Luminal B, *n* = 24; Her2, *n* = 19; TNBC, *n* = 102. Data were plotted as the means of 95% confidence interval ± s.d. **c** Western blot analysis of KTN1 expression. Below shows the densitometric quantification of KTN1 expression relative to MCF10A. Kaplan–Meier analyses of relapse-free survival, post progression survival of breast cancer patients from **d** ‘high’ and ‘low’ *KTN1* mRNA expression from Kaplan-Meier plotter dataset **e** ‘high’ and ‘low’ KTN1 protein expression from Tang_2018 dataset. One-Way ANOVA and Dunnett’s multiple comparison test were used to analyze the data. *P < 0.05, **P < 0.01, ***P < 0.001. Scale bars, 100 µm

To identify essential biomarkers that potentially participate in TNBC tumorigenesis, we analyzed that the gene profiles of 81 basal BCa samples and 62 other subtypes of BCa from The Cancer Genome Atlas (TCGA) dataset using edger analysis.^18^ Gene Ontology (GO) with the molecular function analysis identified top-ranked lists genes (Supplementary Fig. S1). The results suggested that the amplified functional molecular pathways include “cell adhesion molecule binding”, “cadherin binding”, “Rho GTPase binding”, “basal transcription machinery”, and “transcription coactivator binding”. Among these pathways, we found that Kinectin 1 (KTN1) was the only one gene which is upregulated in common (Fig. 1a). Previous studies have demonstrated that KTN1 is located on the endoplasmic reticulum (ER) membranes and interacted with the cargo binding site of kinesin to, which promote its microtubule-stimulated ATPase activity.^4^ Importantly, KTN1 was a downstream effector of microtubule-dependent Rho GTPase, which promoted lysosomal transport.^4^ Constitutively activation of Rho GTPase induced tumor formation in immunocompromised mice.^19^

Immunohistochemistry (IHC) analyses were performed in adjacent tissues, luminal A, luminal B, Her2 positive, and TNBC tissues using two commercial tissue microarrays. The protein levels of KTN1 expression in TNBC tissues were significantly higher than other tissues. Whereas the expression of KTN1 protein from other three subtypes of breast cancers did not show a difference compared to adjacent tissues (Fig. 1b). In addition, Western blot analysis revealed that KTN1 was upregulated in basal BCa cell lines (MDA-MB-231, BT549, and HCC38) compared with the normal breast epithelial cells (MCF10A) and other luminal BCa cell lines (MCF-7, ZR75-1, and T47D) (Fig. 1c).

To explore a role of KTN1 in BCa clinical outcomes, we analyzed the survival of BCa patients using Kaplan–Meier plotter (www.kmplot.com). The results showed that high expression of KTN1 mRNA had a poor relapse-free survival and post progression survival rates (Fig. 1d). Overexpression of KTN1 protein was positively correlated with reduced overall survival (Fig. 1e). The results indicated that KTN1 was upregulated in TNBC tumors and, may play a role in regulating TNBC cell malignancy.

### KTN1 promotes invasive TNBC cell growth

**Fig. 2:**
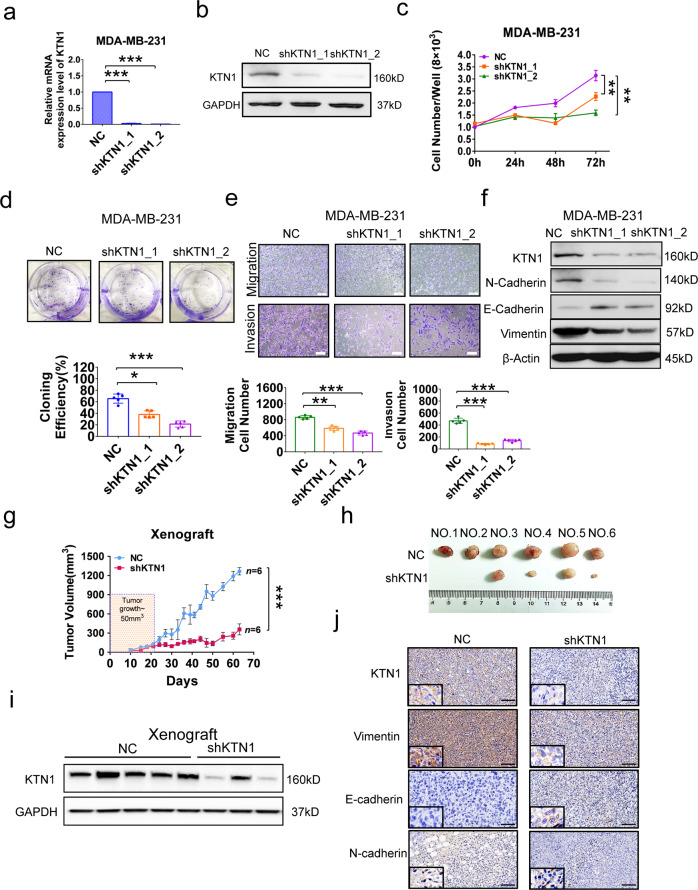
Knockdown of KTN1 inhibited TNBC tumor growth, invasion and EMT both in vitro and in vivo. Knockdown of KTN1 in MDA-MB-231 cell by KTN1_1 and KTN1_2 shRNA vectors was determined by qRT-PCR (**a**) and Western blot assay (**b**) compared with negative control vector (NC). **c** KTN1 knockdown inhibited cell proliferation by CCK-8 assay at 0 h, 24 h, 48 h, and 72 h. **d** KTN1 knockdown reduced cell colony formation by colony formation assay. **e** KTN1 knockdown inhibited cell migration and invasion by Transwell assay. Each experiment was performed in triplicate and data a represented as mean ± s.d. One-Way ANOVA and Dunnett’s multiple comparison test were used to analyze the data (*P < 0.05, **P < 0.01, ***P < 0.001). **f** Western blot analysis on EMT biomarkers in KTN1 knockdown cells. **g** Tumor volume was measured after injected MDA-MB-231 cells with KTN1 shRNA in the xenograft mouse model; *n* = 6, ***P < 0.001. **h** Representative images of tumors in nude mice-bearing MDA-MB-231 cells transfected with NC and KTN1 shRNA; *n* = 6. **i** Western blot assay was performed to detect the protein expression of KTN1. **j** Xenograft tissues were photographed, fixed, and stained with IHC staining

To explore the functional role of KTN1 in TNBC, the expression of KTN1 was inhibited with two shRNA vectors in TNBC cell lines (Fig. 2a-b). The results showed that knockdown of KTN1 significantly reduced the proliferation of TNBC cells (Fig. 2c) and led to decrease cell colony formation efficiency (Fig. 2d). Additionally, KTN1 deficiency significantly impaired the migration and invasion of TNBC cells (Fig. 2e). Given the role of epithelial-mesenchymal transition (EMT) in cancer metastasis mechanisms, we further examined the levels of KTN1 expression and EMT biomarkers in these cells. Western blot assay showed that loss of KTN1 repressed the expression of mesenchymal biomarkers Vimentin, N-cadherin, and increased the epithelial biomarkers E-cadherin (Fig. 2f), indicating that KTN1 might promote the TNBC progression via regulating EMT. Importantly, similar results were obtained from another TNBC cell line using the same loss-of-function analysis (Supplementary Fig. S3a–f).

To confirm the effect of KTN1 in vivo, the cells with KTN1 knockdown were injected into the mammary fat pad of nude mice to establish a BCa xenograft model. Significant difference in xenograft volumes was found in until the experimental endpoint (Fig. 2g). As shown in Fig. 2h, knockdown of KTN1 reduced the primary tumor growth as compared with the negative control groups (NC) after injection of tumor cells at 63 days. Western blot analysis showed that the KTN1 protein expression was significantly decreased (Fig. 2i). Hematoxylin and eosin staining, and IHC staining of tumor tissues were performed. The data showed that the expression of mesenchymal markers was significantly decreased, while epithelial biomarkers was increased (Fig. 2j). These findings revealed that KTN1 was necessary for TNBC tumorigenesis and progression, and that inhibition of KTN1 is equipped to block TNBC growth.

### High-throughput transcriptome sequencing reveals KTN1-mediated signaling pathways in TNBC tumor

**Fig. 3:**
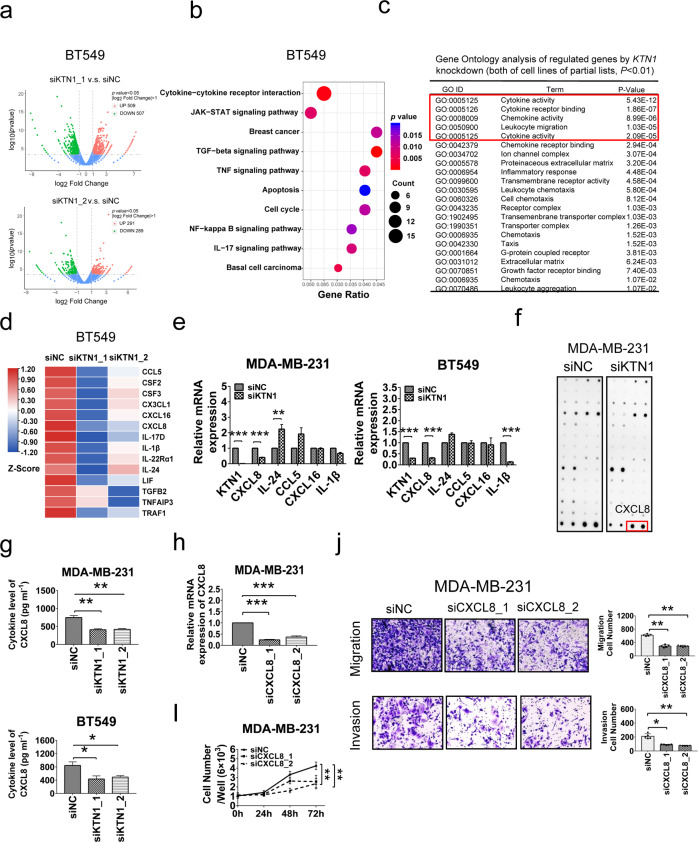
High-throughput transcriptome sequencing analysis of KTN1-regulated downstream gene profile in TNBC cells. **a** Total RNAs were isolated from BT549 cells treated with control oligos and KTN1 siRNA oligos and subjected to RNA-seq. Up- and down-regulated genes were shown by volcano plot. **b** Representative differential signaling pathways were highlighted by KEGG pathway analysis in BT549 cells. **c** Representative Gene Ontology analysis of differentially expressed genes. BP: biological process; MF: molecular function; CC: cellular component; **d** A heat map analysis of representative differential cytokine genes in BT549 cells treated by KTN1 siRNA oligos compared with control oligos. **e** Identification of KTN1 and top six down-regulated genes in siKTN1 oligos-treated cells. **f** Cytokine antibody arrays profiling of cytokine secretions in the supernatant of MDA-MB-231 cells with control oligos or KTN1 siRNA oligos. **g** ELISA assay on the secretion of CXCL8 in MDA-MB-231 cells and BT549 cells transfected with KTN1 siRNA oligos compared with control oligos. **h** QRT-PCR analysis of mRNA expression of CXCL8 in MDA-MB-231 cells transfected with CXCL8 siRNA oligos compared with control oligos. **i** CXCL8 knockdown inhibited MDA-MB-231 cell proliferation at 0 h, 24 h, 48 h, and 72 h by CCK-8 assay. **j** CXCL8 knockdown inhibited cell migration and invasion by Transwell assay. Each experiment was performed in triplicate and data a represented as mean ± s.d. One-Way ANOVA and Dunnett’s multiple comparison test were used to analyze the data (*P < 0.05, **P < 0.01, ***P < 0.001). Scale bars, 100 µm

To further understand the potential mechanisms KTN1 gene in regulating TNBC progression, we analyzed the gene profiles of BT549 and MDA-MB-231 cells treated with siNC or siKTN1 oligos and identified differentially expressed genes through transcript quantification by high-throughput transcriptome sequencing (RNA-seq). The volcano plot analysis of differentially expressed genes with *P*-value < 0.05 revealed that 509 genes were upregulated, and 507 genes were down-regulated in siKTN1_1-treated compared to siNC-treated in BT549 cells; 291 genes were upregulated, and 289 genes were downregulated in siKTN1_2-treated cells (Fig. 3a). Kyoto Encyclopedia of Genes and Genomes (KEGG) pathway analyses identified that the differently expressed genes induced by KTN1 deficiency were involved in the cytokine-cytokine receptor interaction pathway (Fig. 3b, Supplementary Fig. S4a). Importantly, the functional annotation chart from Gene Ontology (GO) revealed that “cytokine activity”, “cytokine receptor binding”, “chemokine activity”, “leukocyte migration”, and “cytokine activity” were classified as the top five GO categories by KTN1 knockdown in two basal BCa cell lines (Fig. 3c, Supplementary Fig. S4a-b). We identified the top five down-regulated genes in siKTN1 oligos-treated cells. We observed that CXCL8 gene was consistently downregulated in these two basal cell lines by qRT-PCR assay in concordance with KTN1 knockdown in both TNBC cell lines (Fig. 3e). Analysis of the 198 patients with basal BCa subtypes from TCGA dataset, based on PAM50 BCa gene signature classifier,^20^ identified that a high KTN1 gene signature was associated with 48 up-regulated cytokines including CXCL8 gene, while a low KTN1 gene signature was significantly associated with downregulation of CXCL8 gene (Supplementary Fig. S4c).

Furthermore, we investigated the potential role of KTN1 knockdown on the secretion function of the CXCL8 in TNBC cells. Human Cytokine Antibody array (RayBio) was performed to detect cytokine profiling using cell supernatant of MDA-MB-231 cells with or without KTN1 knockdown (Fig. 3f). The results showed that cytokines secretion was reduced on KTN1 knockdown, including CXCL8 protein, which was previously shown to be essential for breast cancer growth previously.^21^ Then, we verified that knockdown of KTN1 reduced the secretions of CXCL8 in the supernatant of MDA-MB-231 and BT549 cell lines using enzyme-linked immune-sorbent assay (ELISA) (Fig. 3g). These results these observations indicated that KTN1 may potentially target CXCL8 to regulate TNBC cell growth and invasion.

Furthermore, we identified that CXCL8 was observably downregulated at both mRNA levels in CXCL8 siRNA oligos groups compared with siNC groups in both cell lines (Fig. 3h, Supplementary Fig. S5a). Also, knockdown of CXCL8 inhibited cell proliferation of two TNBC cell lines (Fig. 3i, Supplementary Fig. S5b). In addition, loss of CXCL8 also decreased the migration and invasion of TNBC cells. These results were similar to the effects of KTN1 depletion (Fig. 3j, Supplementary Fig. S5c). Further analysis revealed that BCa patients with the high of CXCL8 expression were linked to a poor overall survival, relapse-free survival, and distant metastasis-free survival rate (Supplementary Fig. S6). These findings suggested that overexpression of CXCL8 might be mediated by KTN1 and contribute to the development of TNBC tumor.

### KTN1 regulates the mRNA expression of CXCL8 via NF-κB/p65 pathway

**Fig. 4:**
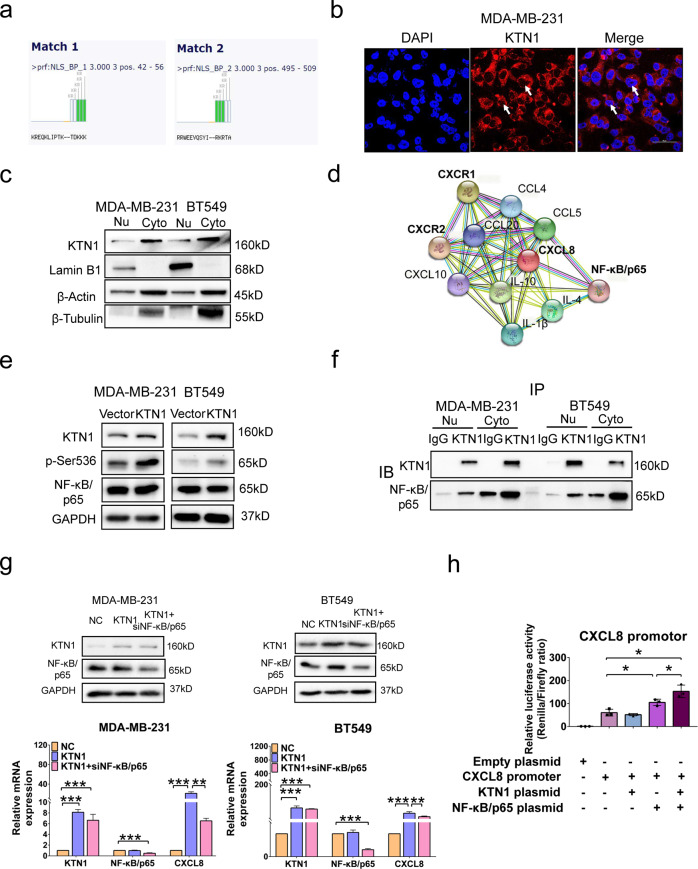
KTN1 regulated CXCL8 mRNA via phosphorylating NF-κB/p65 in TNBC cells. **a** Myhits bioinformatics database (https://myhits.sib.swiss/cgi-bin/motif_scan) analysis of functional domains of KTN1. **b** KTN1 were localized in the nucleus of MDA-MB-231 cells by laser confocal microscopy. **c** Cytoplasm and nuclei protein separation determined with a Western blot assay. Lamin B1 and β-Tubulin were used as nuclear and cytoplasmic biomarkers, β-Actin was the whole-cell biomarker. **d** String bioinformatics database (https://string-db.org/)analysis of the functional interaction of CXCL8 and NF-κB/p65. **e** Western blot analysis of protein expression on KTN1, phosphorylated NF-κB/p65 (Ser536), NF-κB/p65 in MDA-MB-231 and BT549 cells transfected with minicircle KTN1 plasmid compared with control vector plasmid. **f** NF-κB/p65 was verified to be the interacting partner of KTN1 by Co-IP followed with Western blot assay. **g** Western blot and qRT-PCR analysis of mRNA expression of KTN1, NF-κB/p65, and CXCL8 in MDA-MB-231 and BT549 cells with KTN1 overexpression or NF-κB/p65 depletion vectors. **h** Luciferase activity of CXCL8 gene promoter reporters in 293 T cells transfected with KTN1 or NF-κB/p65 plasmid. Each experiment was performed in triplicate and data a represented as mean ± s.d. One-Way ANOVA and Dunnett’s multiple comparison test were used to analyze the data (*P < 0.05, **P < 0.01, ***P < 0.001)

The KTN1 gene is located on the 14th chromosome and encodes the kinectin protein, which binds to kinesin protein to regulate organelle transport.^22^ The KTN1 protein as a key co-factor of RhoG microtubule-dependent cellular activity,^4^ is found to support focal adhesion molecule growth in the lamella of cellular endoplasmic reticulum.^23^ However, using Myhits bioinformatics database (https://myhits.sib.swiss/cgi-bin/motif_scan), we found two novel functional domains of KTN1 with potential nuclear localization signals (NLSs) that were located at regions from 42 to 56 amino acid or from 495 to 509 amino acid. These two NLSs might modulate downstream target pathway of KTN1 gene (Fig. 4a). Imaging of KTN1 protein in MDA-MB-231 cells by confocal laser scanning microscopy showed that it located in the cytoplasm and nucleus (Fig. 4b). Additionally, Western blot assay confirmed that KTN1 expressed in the cytoplasm and nuclei of both TNBC cells (Fig. 4c). These results revealed that KTN1 might regulate CXCL8 expression via KTN1 nuclear translocation.

To explore the molecular mechanism of modulating the transcription of CXCL8, we found that NF-κB/p65 subunit was in the upstream target of CXCL8 by analyzing the String database (https://string-db.org/) and KEGG pathway database (https://www.kegg.jp/kegg/pathway. html) (Fig. 4d). In TNBC, NF-κB pathway was found to be frequently activated via both autocrine and paracrine effects, which resulted in enhancing activation of inflammatory cytokines.^21^ Given that NF-Κb/p65 played a crucial role in the NF-κB signaling pathway, we next investigated whether KTN1 involves in NF-κB/p65-induced CXCL8 expression. Firstly, Western blot analysis identified that the phosphorylated levels of NF-κB/p65 protein (Ser536) were significantly increased in KTN1 overexpressed cells, however, overexpression of KTN1 had no change on the total protein expression of NF-κB/p65 (Fig. 4e). Consistently, knockdown of KTN1 led to decrease the expression of phosphorylated NF-κB/p65 (Ser536) in two cell lines as compared to siNC oligo groups (Supplementary Fig. S7a). These results indicated that KTN1 enhanced the phosphorylation of NF-κB/p65 protein at Ser536 site.

Next, we analyzed whether KTN1 protein regulated the CXCL8 expression through binding to NF-κB/p65 protein. A Co-IP assay was performed using a KTN1 antibody and a mouse IgG as negative control. The KTN1 Co-IP results showed that NF-κB/p65 proteins were able to be pulled down by KTN1 antibody in nucleus and cytoplasm, validating their binding potential in TNBC cells (Fig. 4f). NF-κB is a crucial for transcription, factor of inflammation, and has a pro-oncogenic effect on pre-malignant cells.^24^ A previous study reported that the endogenous CXCL8 promoter is activated by NF-κB-dependent Werner Protein recruitment.^25^ We investigated whether KTN1 overexpression induced NF-κB/p65 protein enrichment. The results suggested that NF-κB/p65 protein and NF-κB/p65(Ser536) protein enrichment was increased in KTN1-overexpressed cells by KTN1, while KTN1 protein and NF-κB/p65(Ser536) protein enrichment was decreased in NF-κB/p65-defiency cells in TNBC cells (Supplementary Fig. S9). These data demonstrated that KTN1 specifically interacted with NF-κB/p65 in TNBC cells.

In addition, to further identify whether KTN1 is a mediator of NF-κB/p65 in activating CXCL8, qRT-PCR analysis was performed. The results revealed that the mRNA expression of CXCL8 were upregulated in KTN1-overexpressed TNBC cells, whereas the mRNA expressions of CXCL8 were reduced by NF-κB/p65 knockdown in KTN1-overexpressed TNBC cells (Fig. 4g). This result was in consistent with the protein secretion of CXCL8 by ELISA assay (Supplementary Fig. S7b). Next, we conducted the luciferase vector containing binding sites of CXCL8, which regulated the expression of luciferase reporter gene to detect the activities of CXCL8 promoter in 293 T cell. As shown in Fig. 4h, CXCL8 was activated after the transfection of overexpressed KTN1 and NF-κB/p65 in 293 T cell compared with NF-κB/p65 plasmid. These results suggested that KTN1 upregulated the mRNA expression of CXCL8 by activating NF-κB/p65 pathway.

### NF-κB/p65 recruits CXCL8 promoter via KTN1 binding sequence

**Fig. 5:**
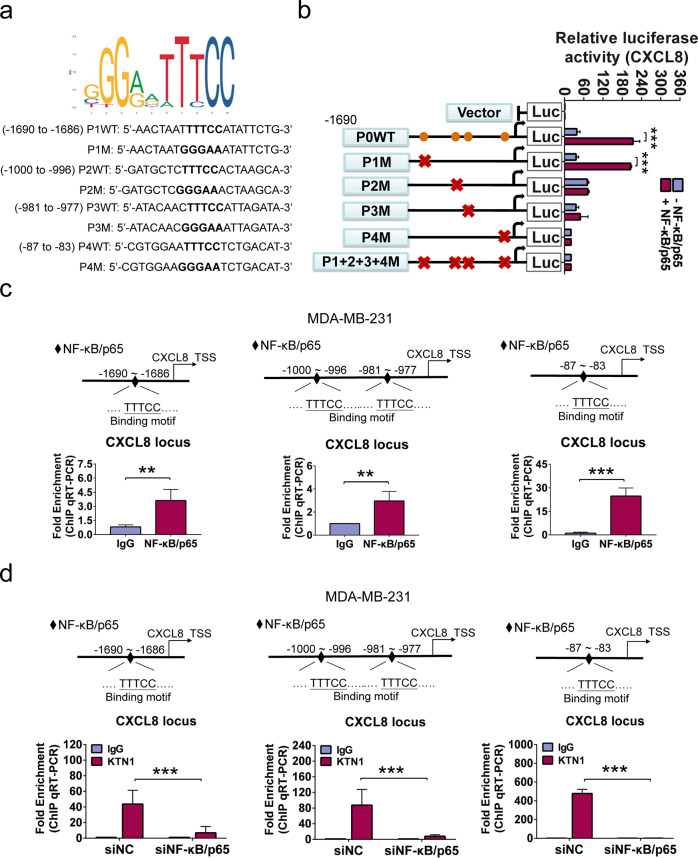
NF-κB/p65 bound the CXCL8 gene to enhance its promoter activity. **a** Predicted binding sites through UCSC genome browser (http://genome.ucsc.edu/) and JASPAR database (http://jaspardev.genereg.net). **b** Luciferase activity of CXCL8 gene promoter reporters in 293 T cells transfected with wild type NF-κB/p65 plasmid or mutant NF-κB/p65 plasmid. Orange circles showed the position of the putative NF-κB/p65-binding sites, and the red “×” showed the mutated NF-κB/p65-binding sites. P, putative binding site; M, mutant; WT, wild type. **c** ChIP analysis of NF-κB/p65 enrichment on promoters of CXCL8 gene in MDA-MB-231 cells. IgG mouse, immunoglobulin G mouse. The different regions containing different putative NF-κB/p65-binding sites from left to right. **d** The binding enrichment of KTN1 at the above binding sites on the promoter regions detected after knockdown of NF-κB/p65. Data shown are mean ± SD of triplicate measurements that have been repeated 3 times with similar results. Data were analyzed using two-tailed Student’s t-test, One-Way ANOVA and Dunnett’s multiple comparison test (*P < 0.05, **P < 0.01, ***P < 0.001)

Previous study identified that CXCL8 expression is regulated via activation of the NF-κB signaling pathway in lung cancer.^26^ To elucidate the interaction of NF-κB/p65 and CXCL8, we searched to approximately 2000 base pairs (bps) of the promoter regions of CXCL8 genes for putative NF-κB/p65 binding sites and constructed the promoter reporters including the predicted binding sites through UCSC genome browser (http://genome.ucsc.edu/) and JASPAR database (http://jaspardev.genereg.net) (Fig. 5a). In 293 T cell, NF-κB/p65 overexpression increased the reporter activity of CXCL8 promoters, whereas mutation of the binding sites 2, 3 or 4 attenuated the activity mediated by NF-κB/p65, and mutation of all binding sites completely inactivated the activity of CXCL8 promoter (Fig. 5b). These data suggested that CXCL8 mRNA expression was specifically activated by NF-κB/p65.

Next, in MDA-MB-231 cells, Chromatin immunoprecipitation (ChIP)-qPCR assay with a NF-κB/p65 antibody confirmed that endogenous NF-κB/p65 proteins were recruited to the binding sites with CXCL8 promoter reporter activity (Fig. 5c). Furthermore, using a KTN1 antibody, we found that KTN1 can also be recruited to CXCL8 promoter. Notably, depletion of NF-κB/p65 using siRNA oligos led to noticeable reduction in the binding of KTN1 at the binding sites of CXCL8 promoter (Fig. 5d). These results revealed that the recruitment of KTN1 protein onto the CXCL8 binding sites was mediated by NF-κB/p65 protein.

### CXCL8 blockade suppresses KTN1-overexpression-induced TNBC tumorigenesis

**Fig. 6:**
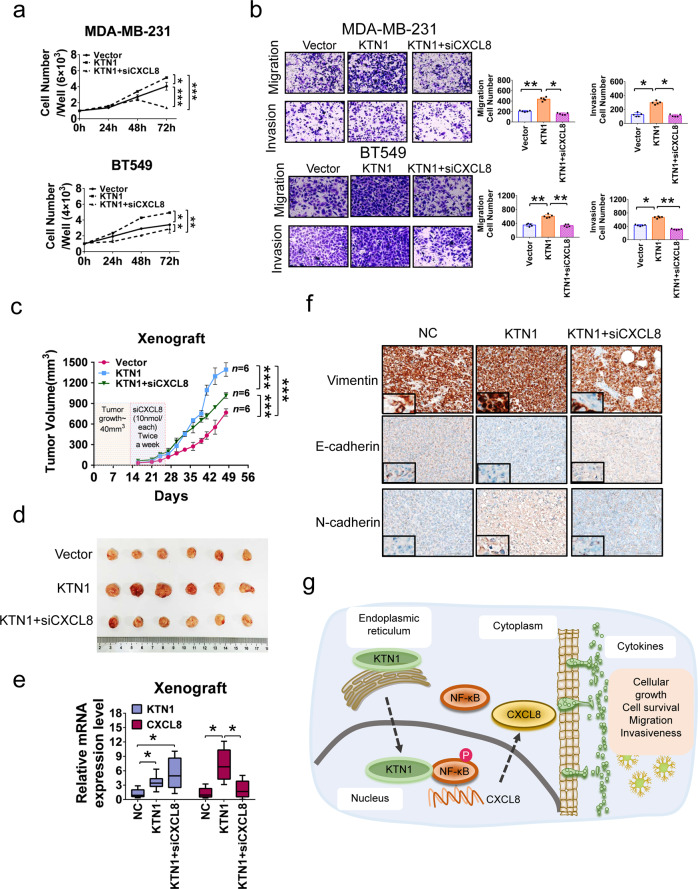
KTN1 was required to promote TNBC tumorigenesis both in vitro and in vivo. **a** CCK-8 assay on cell viability in KTN1 overexpressed MDA-MB-231 and BT549 cells transfected with CXCL8 siRNA oligos. **b** Migration and invasiveness of KTN1 overexpressed MDA-MB-231 and BT549 cells transfected with CXCL8 siRNA oligos determined by Transwell assays. **c** Tumor growth curves show the development of xenograft of MDA-MB-231 cells transfected with KTN1 overexpression or CXCL8 siRNA oligos. **d** The NSG mice were sacrificed at the end of the experiment and the dissected tumors were photographed (*n* = 6). **e** QRT-PCR analysis of the mRNA expression of KTN1 and CXCL8 in xenograft tumors (*n* = 6). **f** Representative images showed the effect of KTN1 overexpression or CXCL8 siRNA oligos on EMT in xenograft. **g** A schematic model of the KTN1-NF-κB-p65-CXCL8-regulatory axis in TNBC development and progression. One-Way ANOVA and Dunnett’s multiple comparison test were used to analyze the data (*P < 0.05, **P < 0.01, ***P < 0.001). Scale bars, 100 µm

IHC analysis on BCa tissue microarrays suggested that KTN1 protein is overexpressed in high-grade BCa compared with other subtypes of BCa (Supplementary Fig. S2), suggesting that upregulation of KTN1 may promote BCa growth and malignancy. Rescue experiments were performed. TNBC cells with KTN1 siRNA-treated were stimulated with CXCL8 protein, which led to a partial rescue of cells growth and invasion compared with the control groups (Supplementary Fig. S8). Moreover, overexpression of KTN1 promoted the proliferation, migration, and invasion of TNBC cells, whereas inhibition of CXCL8 with siRNA oligo partly counteracted the increase of KTN1-overexpressed TNBC cells (Fig. 6a, b).

To identify the pro-carcinogenic role of KTN1 in vivo, we assessed the effects of KTN1 overexpression, and CXCL8 knockdown by siRNA oligos of cholesterol and dimethoxy-modified in KTN1 overexpressed cells on MDA-MB-231 xenograft mammary tumor growth in NOD/SCID/IL2rγ null (NSG) mice. The results showed that the xenograft tumor growth was notably increased in KTN1-overexpressed groups, compared with the vector control groups (*n* = 6). However, the tumor growth was significantly reduced in mice bearing tumors expressing CXCL8 siRNA oligos groups, compared with the KTN1 overexpression groups (*n* = 6) (Fig. 6c). As shown in Fig. 6d, the treatment with CXCL8 siRNA oligos in KTN1-upregulated groups reduced the tumor growth. QRT-PCR analysis showed that KTN1 mRNA levels were significantly increased and CXCL8 mRNA level was obviously decreased in tumors (Fig. 6e). In addition, IHC analysis of the harvested tumors demonstrated that the expression of mesenchymal biomarkers was markedly decreased, whereas epithelial biomarkers were increased (Fig. 6f). Taken together, these findings demonstrated that overexpression of KTN1 promoted TNBC tumorigenesis, whereas, knockdown of CXCL8 could neutralize the effect of KTN1 in vivo.

## Discussion

There is no effective targeted therapy available for the treatment of TNBC owing to the lack of ER, PR, and Her2 receptor expression. In recent years, increasing attentions have been paid to the exploitation of novel targets for the diagnosis and treatment of TNBC. Walid Khaled et al. identified that the transcription factor BCL11A, which had higher expression in TNBC.^27^ In addition, the progression-free survival of patients treated with a combination of the anti-PD-1 antibody pembrolizumab and chemotherapy was improved in a clinical trial with advanced-stage TNBC.^28^ Hence, it is essential to comprehensively exploit the molecular mechanisms involved in TNBC progression and identify reliable prognostic targets.

Itaru Toyoshima first found that the KTN1 protein of the endoplasmic reticulum (ER) is a membrane receptor which interacts with kinesin protein for the microtubule-based organelle transport.^3^ The kinesin-binding domain on KTN1 is located at the COOH terminus and promotes the microtubule-mediated kinesin-ATPase activity. It was suggested that KTN1 was referred to be involved in the regulation of the organelles.^29^ Besides, KTN1 was also involved in regulating protein synthesis via anchoring the translation elongation factor-1 complex in endoplasmic reticulum of eukaryotic cells.^30^ Previous studies revealed that increased expression of KTN1 contributed to tumor cells growth and invasion. In bladder cancer, it was confirmed that lncRNA KTN1 AS1 overexpression promotes the recruitment of a histone acetyltransferase EP300. This process caused upregulation of KTN1, which bound to Rho GTPase to promote tumor progression.^31^ Although these studies showed that KTN1 had associated with cancer progression, the molecular mechanism of KTN1 in cancers, particularly in BCa is unknown. In this study, we found that KTN1 expression was significantly increased in TNBC tissues compared to adjacent tissues and other BCa subtypes, and high KTN1 expression was linked to poor prognosis of TNBC patients. Furthermore, functional studies suggested that overexpression of KTN1 accelerated the proliferation, migration, and invasion of TNBC cells, and loss of KTN1 inhibited the growth of xenograft tumor in vivo. These results indicated that KTN1 plays an oncogenic role in BCa.

KTN1 was a multifunctional protein that interacts with Kinesin, Rho family GTPases, and translation elongation factor-1δ (EF-1δ), KTN1 also participated in many processes of cellular dynamics such as the organelle motility, focal adhesion growth of cellular lamella, and anchoring EF-1δ complex onto ER.^23,29,30^ Previous studies reported that KTN1 acts as a cytoplasmic protein in regulating cellular functions, however, the participation of KTN1 in cellular transcriptional regulation remains poorly understood. In this study, our findings demonstrated that the involvement of KTN1 in transcriptional regulation of downstream target genes and the proinflammatory factor CXCL8. Cytoplasmic gene may involve in modulating protein stability and modification. Tang et al. found that cytoplasmic lncRNA GLCC1 stabilized transcriptional factor c-Myc form ubiquitination by binding to HSP90 partner.^32^ In support of our observation, other studies had shown that tumor suppressor Ring1 and YY1 binding protein (RYBP), which was located cytoplasm, was able to bind to caspase 8, to inhibit MDM2-mediated polyubiquitination and degradation of p53 via the nuclear translocation of RYBP.^33^ Here, we observed that there were two potential NLSs of KTN1 protein domains, it indicated that KTN1 might involve in controlling CXCL8 expression via the nuclear translocation of KTN1.

In this study, we identified that CXCL8 was one of the downstream targets of KTN1. CXCL8 and its receptors (CXCR1/CXCR2) signaling in the microenvironment of promoted BCa and promoted its progression and metastasis.^13^ There are two common types of NF-κB signaling pathway: the canonical and the alternative pathways. The canonical pathway targets p50-p65 (encoded by *RELA*) dimers, but alternative pathway targets p50- *RELA* dimers.^9^ Canonical NF-κB pathway promotes angiogenesis and invasion of cancers by modulating pro-angiogenic factors, involving CXCL8.^34^ Previous studies revealed target gene activation by NF-κB pathway through recruiting other transcription factors bond to target DNA-binding sites, such as signal transducer and activator of transcription (STAT).^35^ According to the String database and KEGG pathway analysis, we investigated that the involvement of KTN1 in transcription regulation of CXCL8, and found it was under the regulation of NF-κB/p65. Overexpressed KTN1 could significantly increase the protein levels of phosphorylated NF-κB/p65 at Ser536 site through interacting with NF-κB/p65. Importantly, we also found that the protein of NF-κB/p65 was increased in KTN1-overexpressed cells by KTN1. These data indicated that KTN1 specifically interacted with NF-κB/p65 in TNBC cells.

In conclusion, we identified a novel gene KTN1 as a carcinogenic promoter in TNBC progression. The overexpression of KTN1 was associated with poor outcome of TNBC patients. Given that HER-3 and HER-4 overexpression is an important prognostic marker in TNBC, their expression profile should be also taken into account for the development of TNBC personalized treatment in patients overexpressing KTN1.^36^ KTN1 protein in cooperation with NF-κB/p65 protein acted a transcriptional activation complex to upregulate the level of proinflammatory cytokine CXCL8. This study contributes a novel mechanism of KTN1 in TNBCs progression and a potential therapeutic target against TNBC.

## Materials and methods

### Breast cancer subtypes and survival analysis

The KTN1 expression data of BCa subtypes samples were obtained from the Cancer Genome Atlas (TCGA) database (http://cancergenome.nih.gov/). The data analysis was performed by R software. A 50 gene signature, PAM50, was used to analyze the biological subtype of BC within the clinical setting.^37^

For Gene Ontology (GO)-molecular function (MF) analysis, the differential analysis of genes was performed by edger analysis between the 81 basal (TNBC) samples of patients and 62 other subtype sample of BCa data.^18^ The significant difference was *P*-value<0.05 and the threshold log_2_[fold change] ≥2.

For interaction of KTN1 and cytokines, KTN1-dependent gene signature was used to identify 198 basal BCa gene expression data in TCGA. Data analysis with high or low KTN1 expression were described as a heat map. Kaplan-Meier survival analyses for the prognosis of breast cancer patients were performed using Kaplan-Meier database (www.kmplot.com).

### Patient samples and immunohistochemistry (IHC)

The BCa tissue sections and tissue microarrays were purchased from Shanghai Outdo Biotech CO., LTD (three commercial tissue microarrays; catalog numbers: #HBreD140Su03, #HBreD077Su01, and #HBreD075Bc01). A total of 206 specimens of BCa (Luminal A 61; Luminal B 24; Her2 positive 19; TNBC 102) and 77 samples from adjacent tissues were included.

For the animal experiments, breast tumor masses were collected and embedded in paraffin after fixing with 4% paraformaldehyde for IHC staining. The IHC staining and evaluation were performed according to the previous report.^5^

### Plasmid constructs, lentiviruses, shRNA, and siRNA

The shRNA sequences of KTN1 were purchased from Sigma-Aldrich and its lentivirus plasmids, virus packaging, and cell infection were performed as described previously.^38^ Virus packaging and cell transfection were performed as described previously.^12^ The promoter of CXCL8 gene was generated and cloned into the pGL3-Basic vector (Promega). The full-length human NF-κB/p65 ORF was generated and cloned into the pCMV-HA vector (Addgene). To construct the parental plasmids for encoding human KTN1, the synthesized full-length coding sequence of human KTN1 was cloned into minicircle vector pMC.BESXP-CMV according to the previous report,^39^ the minicircle encoding human KTN1 was produced in the E. coli strain ZYCY10P3S2T22. The mutated CXCL8 promoter luciferase reporter plasmids were made by recombinant PCR for luciferase reporter assay. The cDNA target sequences of siRNAs (RiboBio CO., LTD) and/or shRNAs for KTN1, CXCL8, and NF-κB/p65 were listed in Table S3.

### Western blot, Enzyme-linked immunosorbent assay (ELISA), and antibodies

Total cell lysates were obtained using RIPA lysis buffer (Thermo) by Western blot analysis was performed as previously described.^40^ The cell nuclear and cytoplasmic extraction assay were performed using NE-PER Nuclear and Cytoplasmic Extraction Reagents kit (Thermo) according to the manufacturer’s instructions. ELISA kit of CXCL8 was purchased from 4 A Biotech CO., LTD (CHE0011) and assay was carried out as per manufacturer’s instructions. The antibodies used in this study were showed in Supporting information.

### Luciferase reporter assay

Luciferase reporter system was purchased from Promega Corporation (Dual-Glo® Luciferase Assay System) and the assay performed according to the manufacturer’s instructions. Briefly, 100 ng wild-type or mutated CXCL8 reporter plasmid, 100 ng KTN1 plasmid, 100 ng NF-κB/p65 plasmid, 100 ng pGL3-Basic and/or 10 ng pRL-SV40 plasmid were transfected with Lipofectamine 3000 (Invitrogen) in 293 T cells. The activity of luciferase and Renilla was determined using the confocal imaging and analysis (BioTek of Agilent, Citation 5 system).

### Co-immunoprecipitation (Co-IP)

MDA-MB-231 and BT-549 cell lysates from cells treated with KTN1 overexpression or negative control vectors were extracted using a Pierce™ Co-Immunoprecipitation Kit (Thermo) according to the manufacturer’s instructions. Briefly, cell lysates were subjected to immunoprecipitation using 20 µg total-KTN1 antibody or 1 μg normal mouse IgG antibody as a negative control. The precipitated protein complex was separated and detected by Western blot assay.

### Chromatin Immunoprecipitation-qPCR (ChIP-qPCR) analysis

ChIP experiments were conducted using an EZ ChIP^TM^ kit (Merck Millipore, 17-371) according to the manufacturer’s instructions. Briefly, cells were cross-linked, pelleted, and the lysis supernatants were sonicated. The sonicated supernatants were incubated with Protein G beads, 10 μg antibody against NF-κB/p65 or 1 μg mouse IgG antibody. The pull-down and purified DNA fragments were detected by qPCR analysis. Relative enrichment was calculated as the amount of amplified DNA normalized to input. Primers were listed in Table S2.

### Tumor xenografts

The xenograft experiments were performed as previously described.^41^ Four- to six-week-old female BALB/c nude mice or female NSG Rag2^−/−^ IL2rg^−/−^ mice were purchased from the Gem Pharmatech (Nanjing, China), and were housed under specific pathogen-free conditions.

For tumor xenograft models, MDA-MB-231 cells (1 × 10^7^ in Matrigel) pretreated with negative control vector or shKTN1 vector were injected into the mammary fat pad of BALB/c nude mice (*n* = 6 in each group).

For rescue experiments, xenograft tumors were established by injection of MDA-MB-231 cells (1 × 10^7^ in Matrigel) into the mammary fat pad of NSG Rag2^−/−^ IL2rg^−/−^ mice. Start from day 0, i.e., three weeks after cancer cells injection, the first group of mice received cells treated with 20 µg KTN1 plasmid in saline diluent, the other two groups received cells treated with the mixture of 20 µg KTN1 plasmid and cholesterol conjugate or CXCL8 siRNAs (RiboBio CO., LTD) (10 nmol) in 50 µl saline once every 3 days for 4 weeks.

The tumor volume was calculated with the following formula: volume (mm^3^) = (length × width^2^)/2. Immediately after the animals were sacrificed, breast tumor masses were collected, fixed in 4% paraformaldehyde and embedded in paraffin. Sections were cut for IHC staining.

### Statistical analysis

All statistical analysis were performed using SPSS 21.0. Correlations between gene expression and clinical indicators of BCa patients were analyzed using Fisher’s exact test. Overall survival, relapse-free survival, and post-progression survival curves were generated by the Kaplan–Meier method and analyzed using the long-rank test. All results are presented as the mean ± standard deviation (sd). Means were compared using independent-sample T test or one-way analysis of variance (ANOVA). All data were analyzed for a normal distribution and homogeneity of variance. *P < 0.05, ** *P* < 0.01, ***P < 0.001.

## Supplementary information

Supplementary data

## Data Availability

All data that support the findings of this study are available from the corresponding author upon reasonable request.
