# Supplementary material for: Kinectin 1 promotes the growth of triple-negative breast cancer via directly co-activating NF-kappaB/p65 and enhancing its transcriptional activity
Source: Signal Transduct Target Ther. 2021 Jul 5;6:250. doi: 10.1038/s41392-021-00652-x (PMC8255318; doi:10.1038/s41392-021-00652-x)
Supplement: Supplementary file 1 — Supplementary data [file 41392_2021_652_MOESM1_ESM.pdf]

Supplementary Materials for

Kinectin 1 promotes the growth of triple-negative breast cancer via directly co-activating NF-kappaB/p65 and enhancing its transcriptional activity

Lin Gao[1,3#], Shanze Chen[4#], Malin Hong[1,6#], Wenbin Zhou[1#], Bilan Wang[9], Junying Qiu[1], Jinquan Xia[1], Pan Zhao[1,6], Li Fu[7], Jigang Wang[1,6], Yong Dai[1], Ni Xie[7], Qinhe Yang[5], Hsien-Da Huang[8, 10], Xiang Gao[2*], Chang Zou[1, 6, 8*]

Chang Zou (zou.chang@szhospital.com), Xiang Gao (xianggao@scu.edu.cn)

This PDF file includes:

Supplementary Methods

Figures. S1 to S9

Report of Human Cell Line Authentication

Supplementary Methods

Ethics statement

The obtained BC tissue arrays was supported by the National Human Genetic Resources Sharing Service Platform (2005DKA21300). This study was approved by the Institutional Review Board of the Second Clinical Medicine College of Jinan University, and all patients provided written informed consent for the use of surgical samples (LL-KY-2019435). Mouse transportation, housing, and breeding were conducted according to recommendations of “The use of non-human animals in research”. Mice were killed by cervical dislocation to prevent suffering. All mouse experiments were conducted in accordance with the ethical standards and guidelines of the Asian Federation of Laboratory Animal Science Associations and were approved by the Second Clinical Medicine College of Jinan University (20200316-23).

Cell Culture

All cell lines were purchased from CellCook Biotech CO., LTD. MCF10A cells were grown in DMEM/F12 medium (Gibco) supplemented with 5% horse serum (Gibco), 10 μg/ml insulin (Gibco), 20 ng/ml epidermal growth factor (EGF), 0.5 μg/ml hydrocortisone. MCF7 cells were grown in MEM medium (Gibco) supplemented with 10% fetal bovine serum (FBS) and 10 μg/ml insulin, 1× nonessential amino acid (CellCook). ZR75-1 and HCC38 cells in RPMI1640 medium supplemented with 10% FBS. T47D and BT459 cells were maintained in RPMI1640 medium supplemented with 10% FBS and 10 μg/ml insulin. MDA-MB-231 cells were maintained in DMEM medium supplemented with 10% FBS. All media were supplemented with 5,000U/ml penicillin/streptomycin (Invitrogen). All cells were maintained at 37°C with 5% CO_2. All cell lines used in this study were authenticated using short tandem repeat typing (results are presented in the Supplementary data 2) and tested to exclude mycoplasma contamination.

Cell Proliferation and Colony Formation Assay

For cell proliferation assay, MDA-MB-231(8000 per well) or BT549 cells (6000 per well) cultivated on 96-well plates (Corning) were transfected with siRNA oligos, and cell proliferation were determined after 0h, 24h, 48h, and 72h by CCK-8 kit (MCE) at 450nm according to the manufacturers' instructions. For cell colony formation assay, MDA-MB-231(1000 per well) or BT549 cells (1000 per well) were cultivated on 6-well plates. Attached cells were transfected with siRNA oligos, and cells were collected at 7 day. Cells were washed twice with PBS and fixed in 4% paraformaldehyde for 1h. Cell colonies were photographed and counted after staining with 0.1% crystal violet.

Cell Migration and Invasiveness

For cell migration assay treated with KTN1 shRNA vectors, KTN1 overexpression with minicircle KTN1 vectors, and CXCL8 siRNA oligos, 1×10^5 cells were seeded in Transwell chambers (Merck Millipore, 8μm) of 24-welll plates (Corning). The complete medium (10% FBS) was added to the bottom of Transwell chambers, while the serum-free medium was added to the top of Transwell chambers. After being cultured between 16h and 20h, the cells were fixed with 4% paraformaldehyde, photographed, and calculated for statistical analysis. Each experiment was repeated at least three times.

For cell invasion assay treated with KTN1 shRNA vectors, KTN1 overexpression with MC KTN1 vectors, and CXCL8 siRNA oligos, 1×10^5 cells were seeded in Matrigel-coated Transwell chambers (BD, 8μm) of 24-welll plates. Other measures were the same as cell migration assay.

For cell migration assay and cell invasion assay treated with KTN1 siRNA oligos or CXCL8 protein, 1×10^5 cells were seeded in Transwell chambers (Merck Millipore, 8μm) of 24-welll plates (Corning), respectively. The complete medium (5% FBS) containing 800 ng/ml CXCL8 protein was added to the bottom of Transwell chambers, while the serum-free medium containing cells was added to the top of Transwell chambers. Other measures were the same as the above contents.

RNA extraction, Quantitative Reverse Transcription-PCR (qRT-PCR), and RNA sequencing

Total RNA was extracted from cells using TRIzol (Life Technologies) according to the manufacturer's instructions. Reverse transcription was conducted using TransScript One-Step RT-PCR SuperMix (Transgen Biotech), and qPCRs were run using SYBR Green PCR Master Mix (Thermo Fisher Scientific) in a in 96-well plate (Bio-Rad). The mRNA relative expression was calculated by the comparative Ct values. Each experiment was repeated at least three times. The primer sequences for qRT-PCR were listed in Table S1. For RNA sequencing, NEB library were prepared to obtain mRNA, subjected to quality control using an Agilent 2100 bioanalyzer. RNA sequencing was performed at Beijing Novogene, using the Illumina Novaseq 6000 platform.

Table S1. Sequences of qRT-PCR primers used in this study.

Primers	
GAPDH	Forward: 5'- ACATCGCTCAGACACCATG-3'
	Reverse: 5'- TGTAGTTGAGGTCAATGAAGGG-3'
β-Actin	Forward: 5'- ACCTTCTACAATGAGCTGCG-3'
	Reverse: 5'- CCTGGATAGCAACGTACATGG-3'
KTN1	Forward: 5'- TGGAATCAGAGCAGAAAAGGG-3'
	Reverse: 5'- GAACTGAAGCGGAGGTCTG-3'
NF-κB/p65	Forward: 5'- AATCCAGTGTGTGAAGAAGC-3'
	Reverse: 5'- GCTGCTCTTCTATAGGAACT-3'
CXCL8	Forward: 5'- ATACTCCAAACCTTTCCACCC-3'
	Reverse: 5'- TCTGCACCCAGTTTTCCTTG-3'
IL-24	Forward: 5'- GAGGAACACGAGACTGAGAG-3'
	Reverse: 5'- TCCAGAGAAGCAGGGTAAAAC-3'
CCL5	Forward: 5'- TGCCCACATCAAGGAGTATTTC-3'
	Reverse: 5'- CCATCCTAGCTCATCTCCAAAG-3
CXCL16	Forward: 5'- CATCTTCATCCTCACCGCAG-3'

	Reverse: 5’- ACCCTCACAAGCTTCCATTC-3
IL-1β	Forward: 5’- ATGCACCTGTACGATCACTG-3’
	Reverse: 5’- ACAAAGGACATGGAGAACACC-3

Western blot, Enzyme-linked immunosorbent assay (ELISA), and antibodies

The following primary antibodies were used: anti-KTN1 [Cell Signaling Technology (CST), #13243, 1:3000], anti-NF-κB/p65 (CST, #8242, 1:3000), anti-phospho-NF-κB/p65 (CST, #3033, 1:1000), anti-β-actin (CST, #3700, 1:3000), anti-GAPDH (CST, #5174, 1:3000), anti-β-Tubulin (CST, #2128S, 1:3000), anti-β-Tubulin (CST, #2128, 1:3000), anti-Lamin B1 (CST, #13435, 1:1000), anti-Vimentin (CST, #5741, 1:3000), anti-E-cadherin (CST, #14472, 1:3000), anti-N-cadherin (CST, #13116, 1:3000). The following secondary antibodies were also used: anti-rabbit IgG-HRP (CST, #7074, 1:2000) and anti-mouse IgG-HRP (CST, #7076, 1:2000); For ChIP and Co-IP assay antibodies: normal mouse IgG (Merck, #12-371). Recombinant Human IL-8 (CXCL8) (PeproTech, 200-08).

Table S2. Sequences of ChIP-qPCR primers used in this study.

Primers	
CXCL8-NF-κB/p65_1	Forward: 5’- CTCAAAGATCATGTTTGTTTGA-3’
	Reverse: 5’- GTAGGAGATGTGAATGCTATG-3’
CXCL8-NF-κB/p65_2	Forward: 5’- GTCCTTGGATAAAGAGCATG-3’
	Reverse: 5’- AGGTATGTTTATGCTCCAGAA-3’
CXCL8-NF-κB/p65_3	Forward: 5’- CTTTCGTCATACTCCGTATTTG-3’
	Reverse: 5’- TCATCACCCTACTAGAGAACTT-3’

Table S3. Sequences of siRNA or shRNA used in this study.

**Sequences**	
siKTN1_1	5’- GAGTGATCTTTCTAGCAAA-3’
siKTN1_2	5’- GAAGTCTGGTGTAATACAA-3’

shKTN1_1	5’-CCGGCCTCTCTAGTTGAAGAACTTACTCGAGTA AGTTCTTCAACTAGAGAGGTTTTTG-3’
shKTN1_2	5’-CCGGCGGTAAACCAACAGCTCACAACTCGAGT TGTGAGCTGTTGGTTTACCGTTTTTG-3’
siCXCL8_1	5’- GTCAGTGCATAAAGACATA-3’
siCXCL8_2	5’- GCCAAGGAGTGCTAAAGAA-3’
siNF-κB/p65_1	5’- CTCAAGATCTGCCGAGTGA-3’
siNF-κB/p65_2	5’- AGACGATCGTCACCGGATT-3’

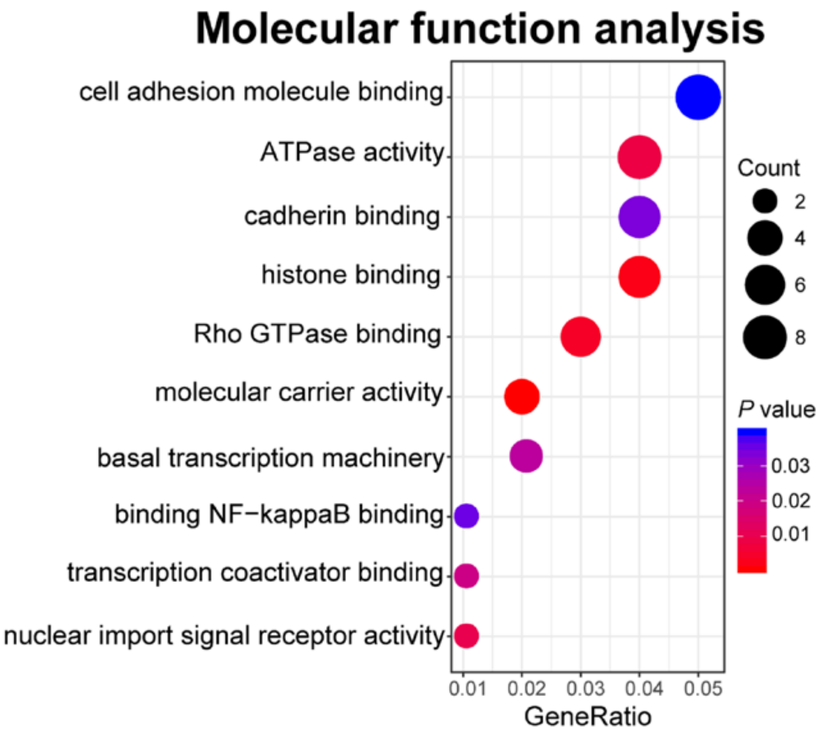

Figure. S1. The top 10 Gene Ontology (GO) analysis with the molecular function of basal BC compared with other subtypes of BC (including Lunimal A, Lunimal B and Her2).

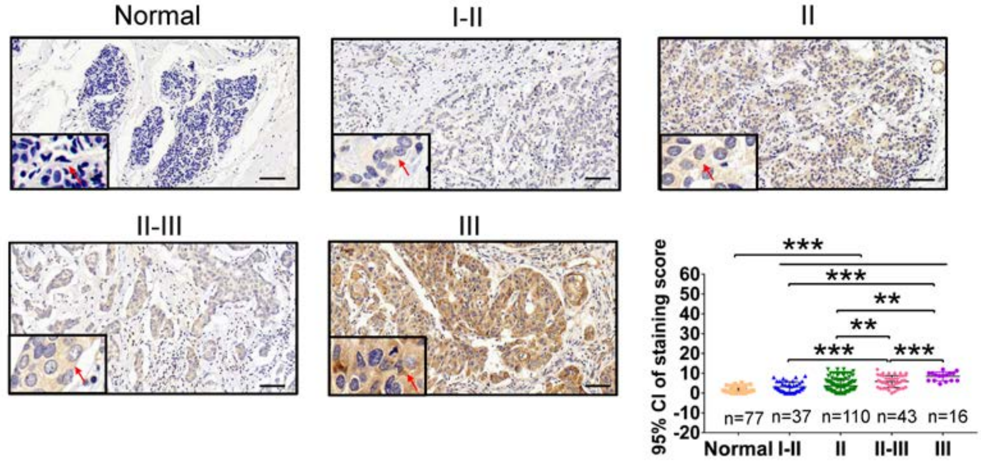

Figure. S2. Association of KTN1 IHC-staining scores with BCs tumor grades (I-II, II, II-III, III). The positive staining was indicated by 'red' arrows. The numbers of cases were showed below. Data were plotted as the means of 95% confidence interval ± s.d. One-Way ANOVA and Dunnett's multiple comparison test were used to analyze the data. *P <0.05, **P < 0.01, ***P < 0.001. Scale bars, 100 μm.

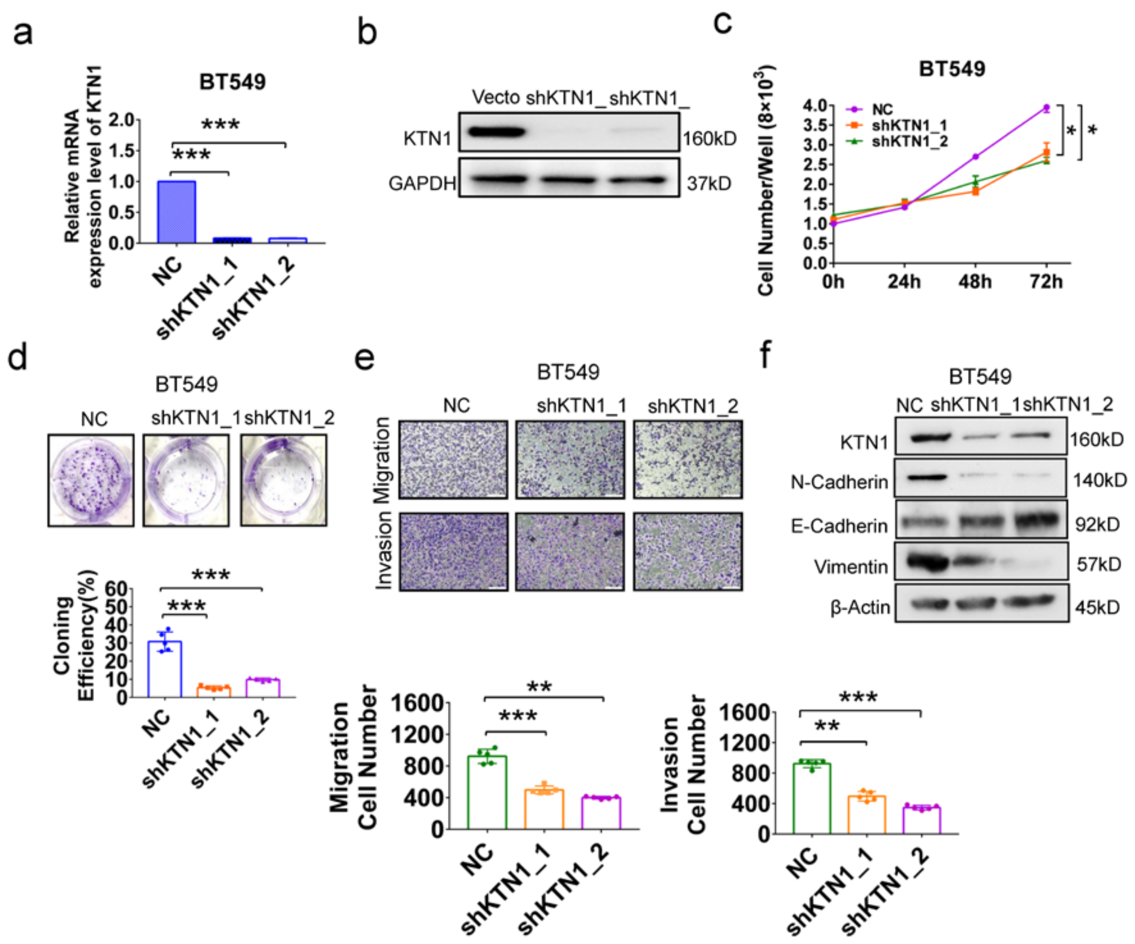

Figure. S3. KTN1 knockdown inhibited the tumorigenesis and invasion of BT549 cells. Knockdown of KTN1 in BT549 cell by KTN1_1 and KTN1_2 shRNA vectors was determined by qRT-PCR. (a) and Western blot assay **(b)** compared with negative control vector (NC). **c** Measurement of cell proliferation by CCK-8 assay cells with NC, shKTN1_1 and shKTN1_2 plasmids treatment. **d** Measurement of cell colony forming efficiency by colony formation assay cells with NC, shKTN1_1 and shKTN1_2 plasmids treatment. **e** Transwell assay analysis of migration and invasiveness cells with NC, shKTN1_1 and shKTN1_2 plasmids treatment. **f** Western blot analysis results of EMT markers in KTN1 knockdown cells. All statistical data represent the average of three independent experiments ±s.d. * *P* < 0.05, ** *P* < 0.01, *** *P* < 0.001. Scale bars, 100 μm.

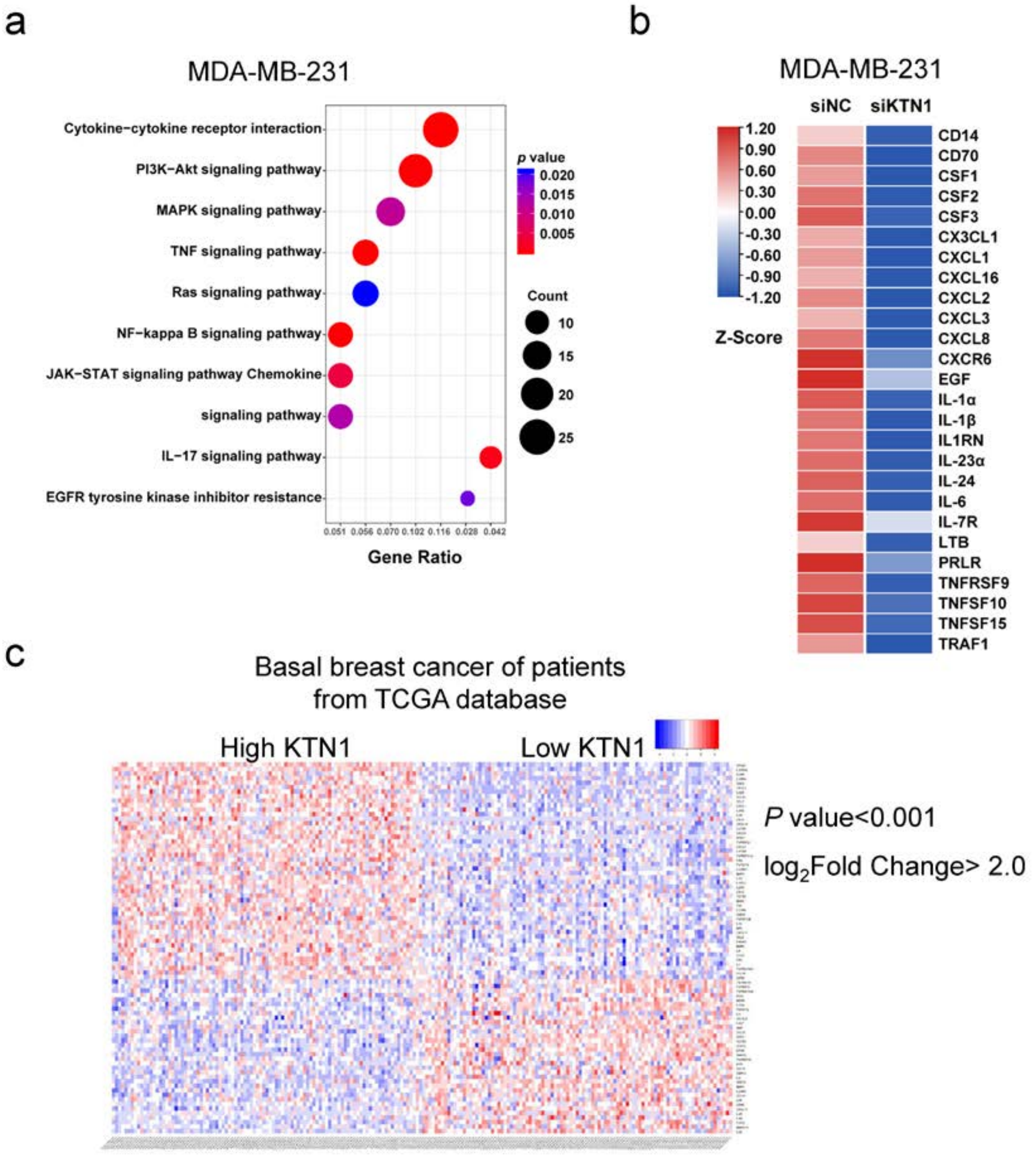

Figure. S4. RNA-seq analysis of KTN1-regulated downstream gene profile changes in TNBC cells. a Represent differential signaling pathways were highlighted by KEGG pathway analysis in MDA-MB-231 cells. **b** A heat map analysis of represent differential cytokine genes in BT549 cells treated by KTN1 siRNA oligos compared with control oligos. **c** Analysis of the 209 basal breast cancer subtypes of patients from TCGA dataset, based on PAM50 breast cancer gene signature classifier. All groups were stratified into ‘High’ and ‘Low’ *KTN1* expression based on autoselect best cutoff. *P*<0.001.

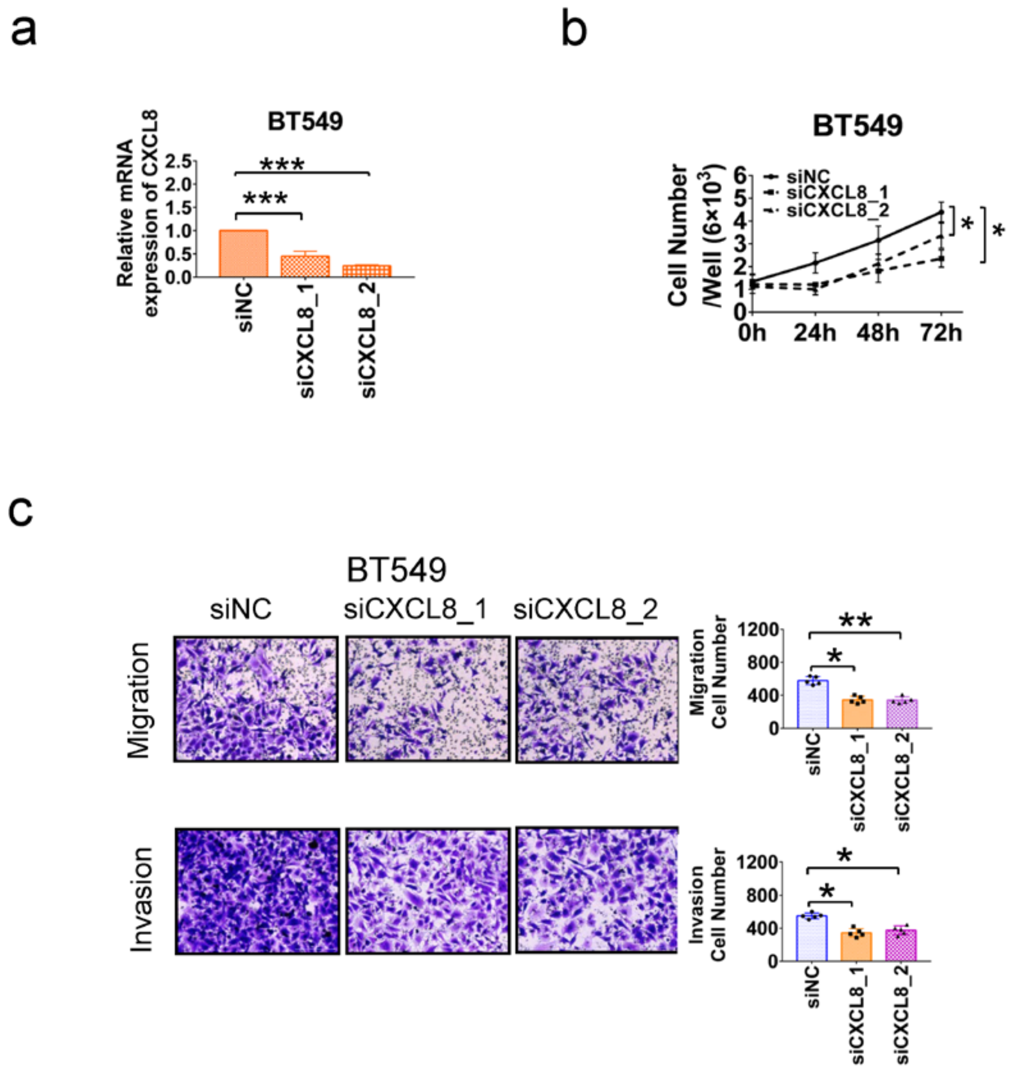

Figure. S5. Knockdown of CXCL8 inhibited BT549 tumor growth and invasion. **a** QRT-PCR analysis of RNA expression of CXCL8 in BT549 cells by CXCL8 siRNA oligos compared with control oligos. **b** CXCL8 knockdown inhibited MDA-MB-231 cell proliferation by CCK-8 assay at 0h, 24h, 48h and 72h. **c** CXCL8 knockdown inhibited cell migration and invasion by Transwell assay. Each experiment was performed in triplicate and data a represented as mean ± s.d. One-Way ANOVA and Dunnett's multiple comparison test were used to analyze the data (*P < 0.05, **P < 0.01, ***P < 0.001). Scale bars, 100 μm.

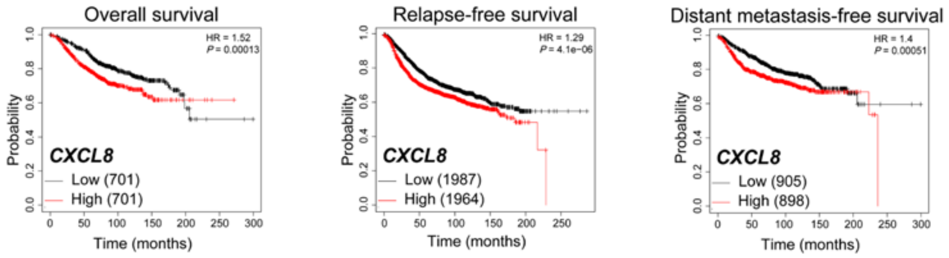

Figure. S6. High CXCL8 mRNA expression revealed a shroter overall survival, relapse-free survival, and distant metastasis-fress survival in BC patients from Kaplan-Meier plotter dataset.

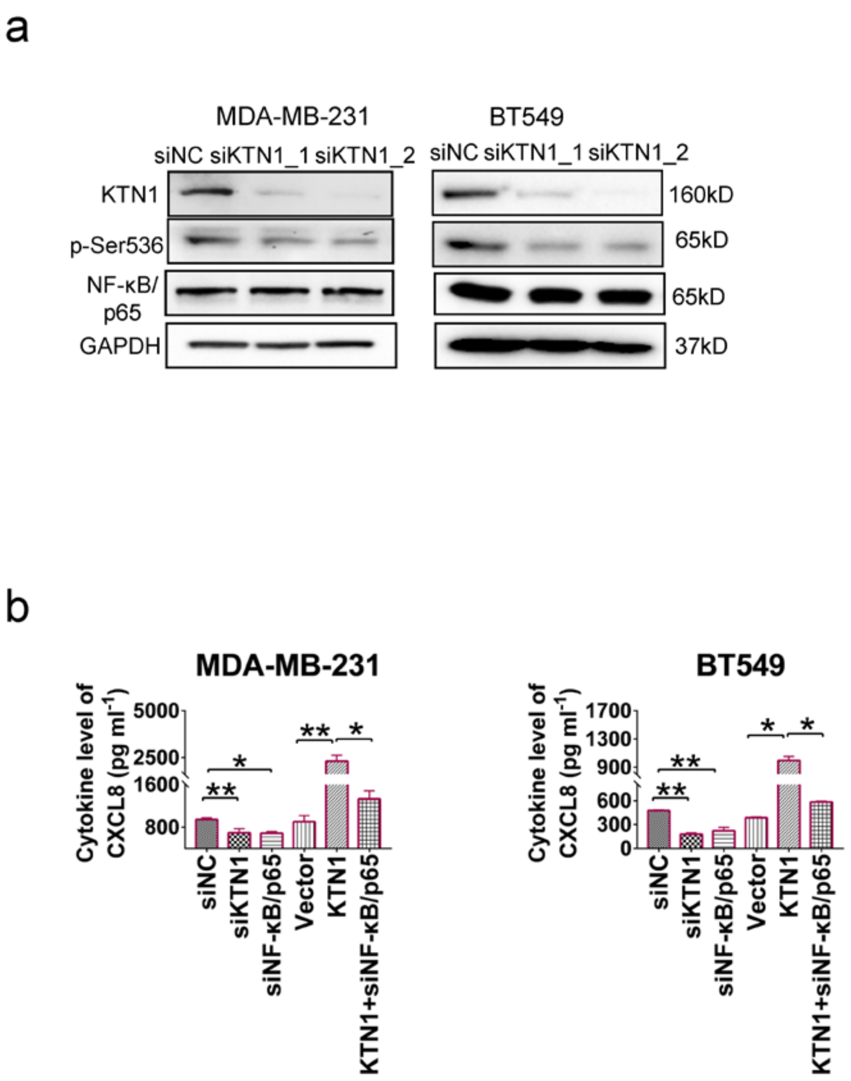

Figure. S7. KTN1 regulated CXCL8 mRNA via phosphorylating NF-κB/p65 pathway in TNBC cells. a Western blot analysis of protein expression of KTN1, phosphorylated NF-κB/p65 (Ser536), NF-κB/p65 in MDA-MB-231 and BT549 cells transfected with NC siRNA oligo, KTN1_1 and KTN1_2 siRNA oligos, respectively. **b** ELISA assay showing the secretion of CXCL8 in MDA-MB-231 cells and BT549 cells transfected with negative control oligos, KTN1 siRNA oligos, NF-κB/p65 siRNA oligos or minicircle KTN1. Each experiment was performed in triplicate and data a represented as mean ± s.d. One-Way ANOVA and Dunnett's multiple comparison test were used to analyze the data (*$P < 0.05$, **$P < 0.01$, ***$P < 0.001$).

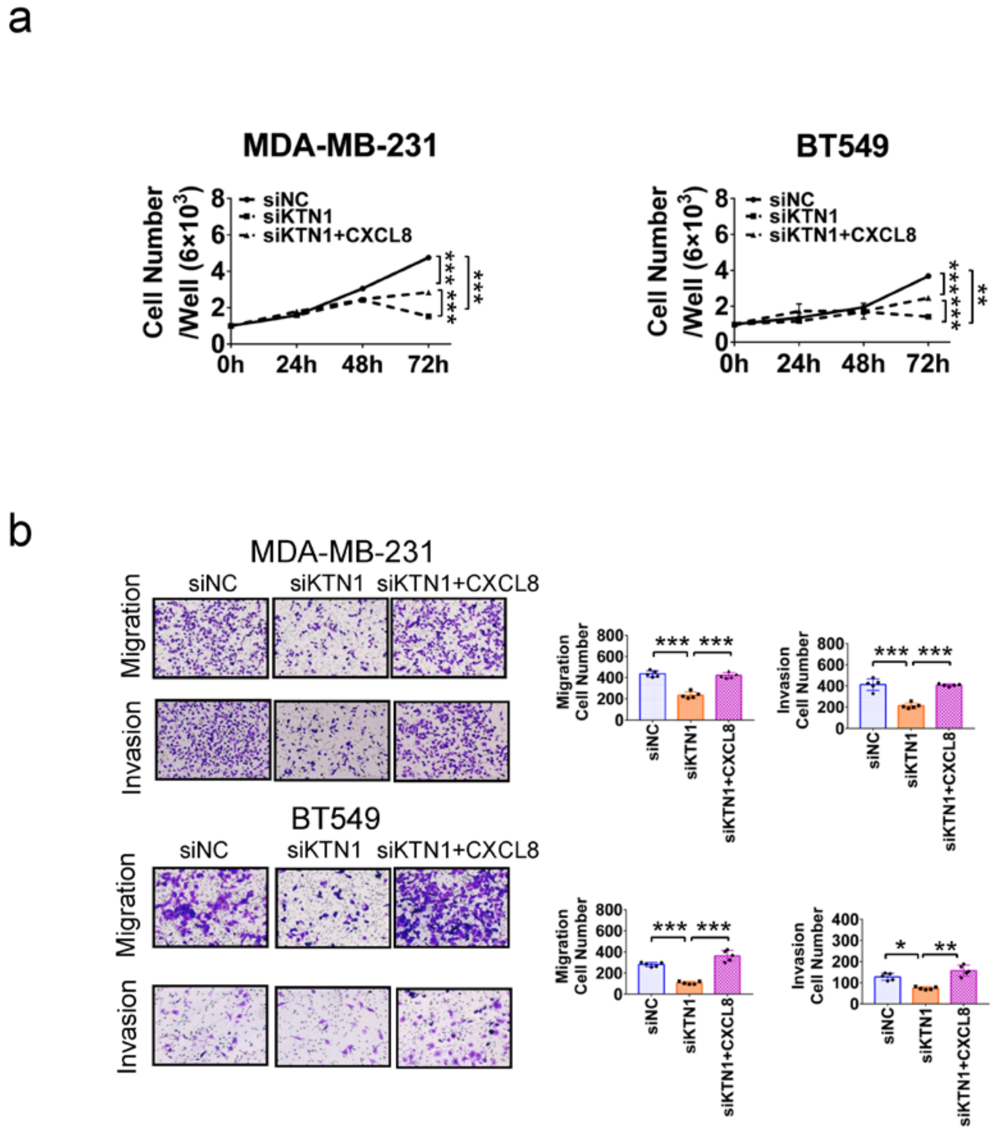

Figure. S8. Rescue experiments by restoring CXCL8 expression after KTN1 depletion re-increased the TNBC cell growth and invasion. a Proliferation analysis of KTN1 depleted MDA-MB-231 and BT549 cells added with CXCL8 protein as determined by a CCK-8 assay. **b** Migration and invasiveness of KTN1 depleted MDA-MB-231 and BT549 cells added with CXCL8 protein as determined by Transwell assays. Each experiment was performed in triplicate and data a represented as mean ± s.d. One-Way ANOVA and Dunnett's multiple comparison test were used to analyze the data (*$P < 0.05$, **$P < 0.01$, ***$P < 0.001$). Scale bars, 100 μm.

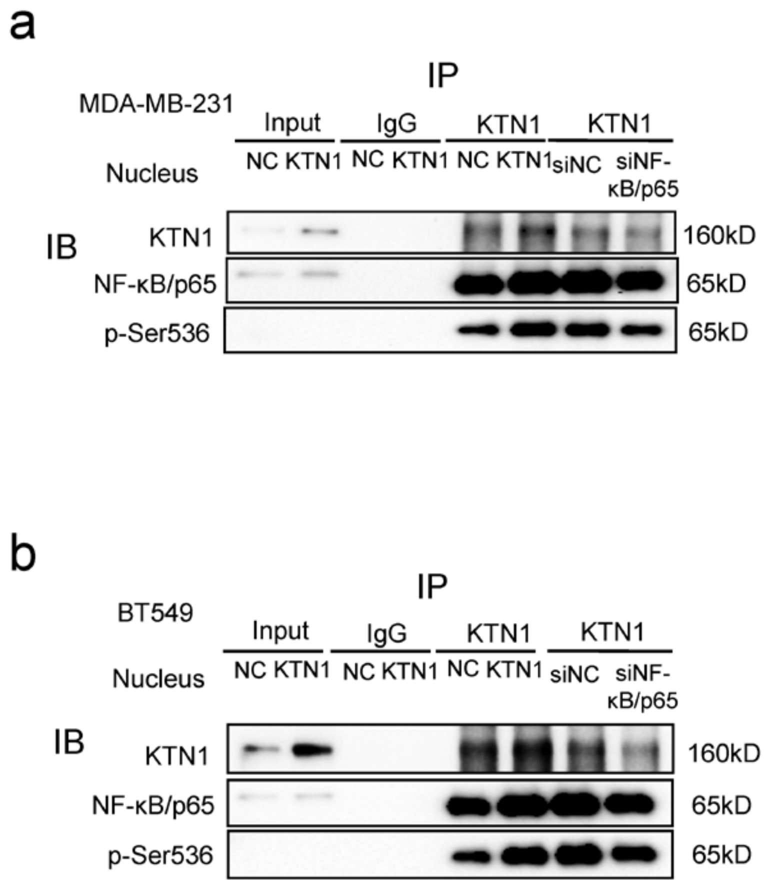

Figure. S9. Co-IP assay and western blot analysis of both KTN1, NF-κB/p65 proteins using specific antibodies was performed to confirm their direct interactions.

Report of Human Cell Line Authentication

（Notice:This authentication report is restricted to the cell sold from Guangzhou Cellcook Biotech Co., Ltd, and the date with seal is the date of delivery. ）

Ⅰ. Sample

Sample Name: labeled as 'MCF-10A'

Ⅱ. Method and Procedure

1. PCR is amplified with STR Multi-amplification Kit (PowerPlex™16HS System);
2. PCR products are assayed with 3100 DNA Analyzer (Applied Biosystems®).
3. Amplification of gene COX1 and electrophoresis are employed to survey the species of the sample.

Ⅲ. Results

1. The STR profiles of the cell line sample are in the attached table and figure.
2. The search result in ATCC and DSMZ databases.
3. The electrophoresis figure of gene COX1.

MCF-10A: ①No loci has tri-alleles or tetra-alleles. Contamination of other human cell line is not found (Figure 1 & Table 1). ②100% matched cell lines are found in ATCC and DSMZ data banks. And the cell line named as "MCF-10A" et *al.* (Figure 2 & Figure 3).③The sample is a human cell line. Contamination of other species cells are not found in the sample (Figure 4).

Operator: Xiaohua Mo　　　　Auditor: Xuanyi Liang

GUANGZHOU CELLCOOK BIOTECH CO.,LTD 17 OCT 2018 CELL STR PROFILING PASS

Guangzhou Cellcook Biotech Co., Ltd

Figure 1. STR profiles of MCF-10A cell line

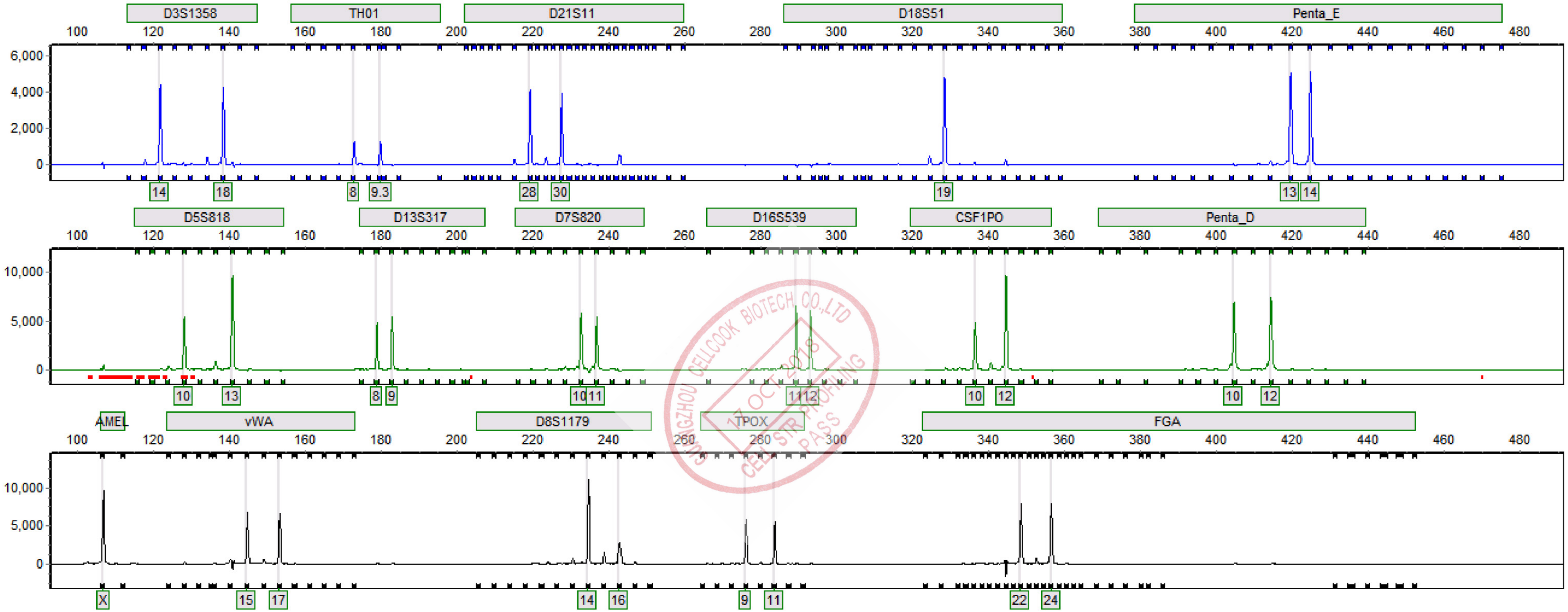

Table 1. STR profiles of MCF-10A cell line

	Allele1	Allele2
D3S1358	14	18
TH01	8	9.3
D21S11	28	30
D18S51	19	
Penta_E	13	14
D5S818	10	13
D13S317	8	9
D7S820	10	11
D16S539	11	12
CSF1PO	10	12
Penta_D	10	12
AMEL	x	
vWA	15	17
D8S1179	14	16
TPOX	9	11
FGA	22	24

BIOTECH CO.,LTD
17 OCT 2018
STR PROFILING
PASS

Figure 2. Search result in ATCC database

SEARCH THE STR DATABASE

As part of our continuing efforts to characterize and authenticate the cell lines in the Cell Biology collection, ATCC has developed a comprehensive database of short tandem repeat (STR) DNA profiles for all of our human cell lines. View our brief tutorial before starting.

1. STR Profiling Analysis
2. Matching Algorithm
3. Interrogating the Database

Showing 1 - 5 Of 5 PageSize: 100

Add to Cart	%Match	ATCC® Number	Designation	D5S818	D13S317	D7S820	D16S539	vWA	TH01	AMEL	TPOX	CSF1PO
☐	81.82	HTB-43	FaDu	12	8,9	11,12	11	15,17	8		11	12
☐	100	CRL-10317	MCF-10A	10,13	8,9	10,11	11,12	15,17	8,9.3	X	9,11	10,12
☐	100	CRL-10318	MCF-10F	10,13	8,9	10,11	11,12	15,17	8,9.3	X	9,11	10,12
☐	100	CRL-10781	MCF-10-2A	10,13	9	10,11	11,12	15,17	8	X	9,11	10,12
☐	81.82	CRL-3212	2A3	12	8,9	11,12	11	15,17	8		11	12

Add to Cart Export to Excel

Figure 3. Search result in DSMZ database

Result of STR matching analysis by your data.

- DSMZ Profile Database -

A graphical presentation is shown at the bottom of this page.

EV	Cell No.	Cell name	Locus names									Figures
			D5S818	D13S317	D7S820	D16S539	VWA	TH01	AM	TPOX	CSF1PO	
	Query (Your Cell)		*10,13*	*8,9*	*10,11*	*11,12*	*15,17*	*8,9.3*	*X*	*9,11*	*10,12*	
1.03(36/35)	CRL-10317	MCF 10A	10,13	8,9	10,11	11,12	15,17	8,9.3	X,X	9,11	10,12	-
1.03(36/35)	CRL-10318	MCF 10F	10,13	8,9	10,11	11,12	15,17	8,9.3	X,X	9,11	10,12	-
1.03(36/35)	CRL-10781	MCF-10-2A	10,13	9,9	10,11	11,12	15,17	8,8	X,X	9,11	10,12	-
1.03(36/35)	CRL-10780	MCF-10-2F	10,13	8,8	10,11	11,12	15,17	8,8	X,X	9,11	10,12	-
0.86(30/35)	CRL-5945	NCI-H2444 [H2444]	10,10	11,12	8,10	12,12	17,17	9.3,9.3	X,X	11,11	12,12	-
0.80(28/35)	439	CAL-72	13,13	8,8	11,11	10,10	15,17	8,8	X,Y	11,11	10,11	-
0.80(28/35)	658	UPCI-SCC-026	13,13	8,11	10,10	11,12	15,18	9.3,9.3	X,X	8,9	10,13	-
0.80(28/35)	CCL-212	MRC-9	12,13	8,11	10,11	12,12	16,17	6,8	X,X	11,11	12,12	-
0.80(28/35)	CRL-2983	UACC-3199	13,13	11,11	10,10	12,12	15,17	9.3,9.3	X,X	10,13	12,12	-
0.80(28/35)	HTB-127	MDA-MB-330	13,13	9,9	10,11	9,12	14,16	8,9.3	X,X	8,11	10,10	-
0.80(28/35)	IFO50072	MRC-9	12,13	8,11	10,11	12,12	16,17	6,8	X,X	11,11	12,12	-

Figure 4. Authentication of the species of the sample

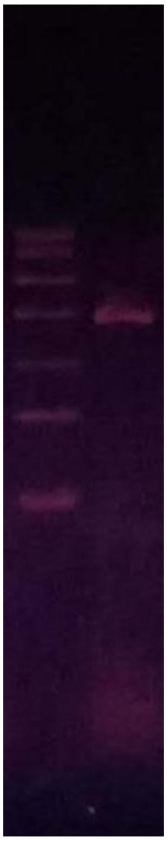

M: Marker. As the size of 700, 600, 500, 400, 300, 200 and 100bp from up to down.

Nine species are checked, as follow: *Homo sapiens* 391bp, *Cricetulus griseus*315bp, *Macaca mulatta*287bp, *Cercopithecus aethiops*222bp, *Rattus norvegicus*196bp, *Canis familiaris*172bp, *Mus musculus*150bp, *Bos Taurus*102bp, IC 70bp

The sample: The band size is 391bp which matches the size of human.

Report of Human Cell Line Authentication

（Notice:This authentication report is restricted to the cell sold from Guangzhou Cellcook Biotech Co., Ltd, and the date with seal is the date of delivery. ）

Ⅰ. Sample

Sample Name: labeled as 'MCF7'

Ⅱ. Method and Procedure

1. PCR is amplified with STR Multi-amplification Kit (PowerPlex™16HS System);
2. PCR products are assayed with 3100 DNA Analyzer (Applied Biosystems®).
3. Amplification of gene COX1 and electrophoresis are employed to survey the species of the sample.

Ⅲ. Results

1. The STR profiles of the cell line sample are in the attached table and figure.
2. The search result in ATCC and DSMZ databases.
3. The electrophoresis figure of gene COX1.

MCF7: ①No loci has tri-alleles or tetra-alleles. Contamination of other human cell line is not found (Figure 1 & Table 1). ②100% matched cell lines are found in ATCC and DSMZ data banks. And the cell line named as "MCF7" *et al.* (Figure 2 & Figure 3). ③The sample is a human cell line. Contamination of other species cells are not found in the sample (Figure 4).

Operator: Xiaohua Mo　　　　Auditor: Xuanyi Liang

Guangzhou Cellcook Biotech Co., Ltd

GUANGZHOU CELLCOOK BIOTECH CO.,LTD
[illegible] JUN 2018
CELL STR PROFILING
PASS

Figure 1. STR profiles of MCF7 cell line

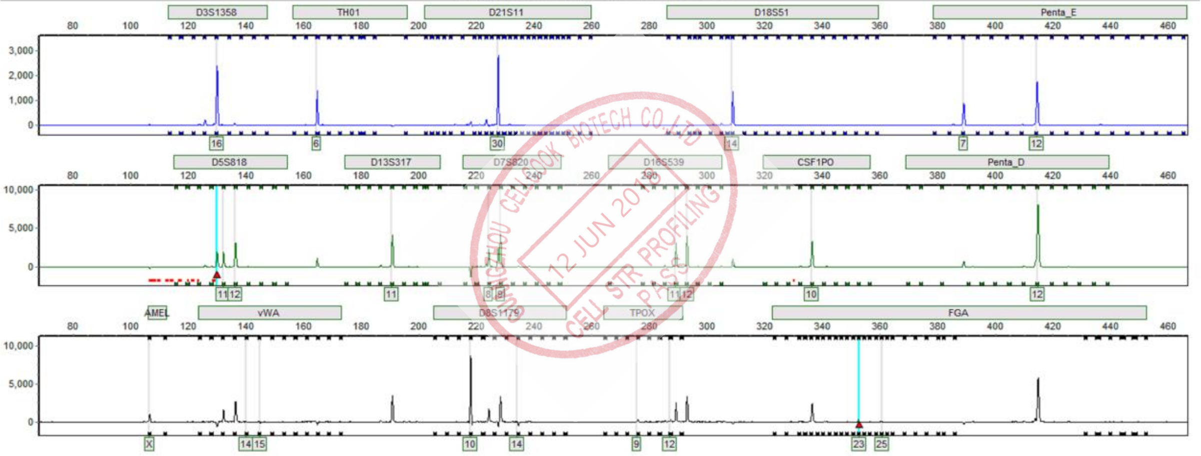

Table 1. STR profiles of MCF7 cell line

	Allele1	Allele2
D3S1358	16	
TH01	6	
D21S11	30	
D18S51	14	
Penta_E	7	12
D5S818	11	12
D13S317	11	
D7S820	8	9
D16S539	11	12
CSF1PO	10	
Penta_D	12	
AMEL	x	
vWA	14	15
D8S1179	10	14
TPOX	9	12
FGA	23	25

Figure 2. Search result in ATCC database

SEARCH THE STR DATABASE

As part of our continuing efforts to characterize and authenticate the cell lines in the Cell Biology collection, ATCC has developed a comprehensive database of short tandem repeat (STR) DNA profiles for all of our human cell lines. View our brief tutorial before starting.

1. STR Profiling Analysis
2. Matching Algorithm
3. Interrogating the Database

Showing 1 - 6 Of 6 PageSize: 100

Add to Cart	%Match	ATCC® Number	Designation	D5S818	D13S317	D7S820	D16S539	vWA	TH01	AMEL	TPOX	CSF1PO
☐	100	HTB-22	MCF7	11,12	11	8,9	11,12	14,15	6	X	9,12	10
☐	100	HTB-22	MCF7/HT29	11,12	11	8,9	11,12	14,15	6	X	9,12	10
☐	81.82	CRL-5900	NCI-H1869	11	11	8,9	12	16	6	X	8,9	10
☐	100	NCIPBCF-1001	MCF7-B7-TS	11,12	11	8,9	11,12	14,15	6	X	9,12	10
☐	100	NCIPBCF-1002	MCF7-G11-TR1	11,12	11	8,9	11,12	14,15	6	X	9,12	10
☐	100	NCIPBCF-1003	MCF7-G11-TR5	11,12	11	8,9	11,12	14,15	6	X	9,12	10

Add to Cart Export to Excel

Figure 3. Search result in DSMZ database

Result of STR matching analysis by your data.

- DSMZ Profile Database -

A graphical presentation is shown at the bottom of this page.

EV	Cell No.	Cell name	Locus names									Figures
			D5S818	D13S317	D7S820	D16S539	VWA	TH01	AM	TPOX	CSF1PO	
	Query (Your Cell)		11,12	11	8,9	11,12	14,15	6	X	9,12	10	
1.12(36/32)	115	MCF-7	11,12	11,11	8,9	11,12	14,15	6,6	X,X	9,12	10,10	-
1.12(36/32)	HTB-22	MCF7	11,12	11,11	8,9	11,12	14,15	6,6	X,X	9,12	10,10	-
1.12(36/32)	JCRB0134	MCF-7	11,12	11,11	8,9	11,12	14,15	6,6	X,X	9,12	10,10	-
1.12(36/32)	RCB1904	MCF7	11,12	11,11	8,9	11,12	14,15	6,6	X,X	9,12	10,10	-
1.06(34/32)	317	KPL-1	11,12	10,11	8,9	11,12	14,15	6,6	X,X	9,12	10,10	-
0.94(30/32)	CRL-5900	NCI-H1869 [H1869]	11,11	11,11	8,9	12,12	16,16	6,6	X,X	8,9	10,10	-
0.81(26/32)	203	SK-N-MC	11,11	11,11	8,8	12,12	17,18	9.3,9.3	X,X	9,11	10,10	-
0.81(26/32)	254	BEN	12,12	12,12	12,12	11,11	15,15	6,6	X,X	8,9	10,10	-
0.81(26/32)	377	TCC-SUP	12,12	11,11	8,9	9,11	14,16	6,9.3	X,X	8,8	10,10	-
0.81(26/32)	CRL-2267	BE(2)-M17	12,12	11,11	9,10	11,11	18,18	6,6	X,X	8,11	10,10	-
0.81(26/32)	CRL-2270	MC-IXC	11,11	11,11	8,8	12,12	17,18	9.3,9.3	X,X	9,11	10,10	-
0.81(26/32)	CRL-7721	MB 157	12,12	11,11	10,11	11,11	15,15	7,8	X,X	9,11	10,10	-

Figure 4. Authentication of the species of the sample

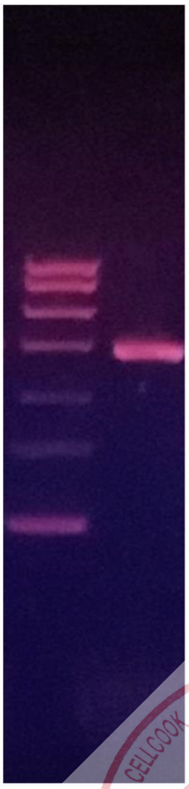

M: Marker. As the size of 700, 600, 500, 400, 300, 200 and 100bp from up to down.

Nine species are checked, as follow: *Homo sapiens* 391bp, *Cricetulus griseus*315bp, *Macaca mulatta*287bp, *Cercopithecus aethiops*222bp, *Rattus norvegicus*196bp, *Canis familiaris*172bp, *Mus musculus*150bp, *Bos Taurus*102bp, IC 70bp

The sample: The band size is 391bp which matches the size of human.

Report of Human Cell Line Authentication

（Notice:This authentication report is restricted to the cell sold from Guangzhou Cellcook Biotech Co., Ltd, and the date with seal is the date of delivery. ）

Ⅰ. Sample

Sample Name: labeled as ‘T47D’

Ⅱ. Method and Procedure

1. PCR is amplified with STR Multi-amplification Kit (PowerPlex^{TM}16HS System);
2. PCR products are assayed with 3100 DNA Analyzer (Applied Biosystems®).
3. Amplification of gene COX1 and electrophoresis are employed to survey the species of the sample.

Ⅲ. Results

1. The STR profiles of the cell line sample are in the attached table and figure.
2. The search result in ATCC and DSMZ databases.
3. The electrophoresis figure of gene COX1.

T47D: ①No loci has tri-alleles or tetra-alleles. Contamination of other human cell line is not found (Figure 1 & Table 1). ②100% matched cell lines are found in ATCC and DSMZ data banks. And the cell line named as “T47D” *et al*. (Figure 2 & Figure 3). ③The sample is a human cell line. Contamination of other species cells are not found in the sample (Figure 4).

Operator: Xiaohua Mo

Auditor: Xuanyi Liang

Guangzhou Cellcook Biotech Co., Ltd

GUANGZHOU CELLCOOK BIOTECH CO.,LTD
12 JUN 2018
CELL STR PROFILING
PASS

Figure 1. STR profiles of T47D cell line

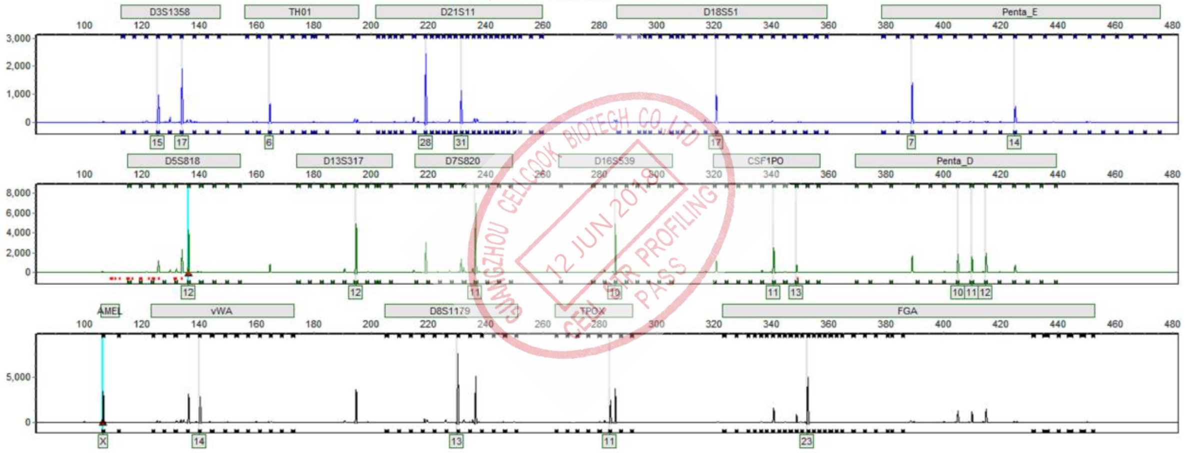

Table 1. STR profiles of T47D cell line

	Allele1	Allele2
D3S1358	15	17
TH01	6	
D21S11	28	31
D18S51	17	
Penta_E	7	14
D5S818	12	
D13S317	12	
D7S820	11	
D16S539	10	
CSF1PO	11	13
Penta_D	10	12
AMEL	x	
vWA	14	
D8S1179	9	12
TPOX	11	
FGA	23	

GUANGZHOU CELLCOOK BIOTECH CO.,LTD
12 JUL 2018
CELL STR PROFILING PASS

Figure 2. Search result in ATCC database

SEARCH THE STR DATABASE

As part of our continuing efforts to characterize and authenticate the cell lines in the Cell Biology collection, ATCC has developed a comprehensive database of short tandem repeat (STR) DNA profiles for all of our human cell lines. View our brief tutorial before starting.

1. STR Profiling Analysis
2. Matching Algorithm
3. Interrogating the Database

Showing 1 - 2 Of 2

PageSize: 100

Add to Cart	%Match	ATCC® Number	Designation	D5S818	D13S317	D7S820	D16S539	vWA	TH01	AMEL	TPOX	CSF1PO
☐	100.0	HTB-133	T-47DBreast CancerHuman	12	12	11	10	14	6	X	11	11,13
☐	100.0	CRL-2865	T47D-KBlucBreast Ductal CarcinomaHuman	12	12	11	10	14	6	X	11	11,13

Add to Cart | Export to Excel

Disclaimer: Reference to this database and the data contained therein may be cited in publications, and ATCC encourages such citation or reference. While every reasonable effort has been made to assure the accuracy of these data, no warranty, express or implied, is made by ATCC as to their accuracy.

Figure 3. Search result in DSMZ database

Result of STR matching analysis by your data.

- DSMZ Profile Database -

A graphical presentation is shown at the bottom of this page.

EV	Cell No.	Cell name	Locus names									Figures
			D5S818	D13S317	D7S820	D16S539	VWA	TH01	AM	TPOX	CSF1PO	
	Query (Your Cell)		12	12	11	10	14	6	x	11	11,13	
1.29(36/28)	737	T47D	12,12	12,12	11,11	10,10	14,14	6,6	X,X	11,11	11,13	-
1.29(36/28)	CRL-2865	T47D-KBluc	12,12	12,12	11,11	10,10	14,14	6,6	X,X	11,11	11,13	-
1.29(36/28)	HTB-133	T-47D	12,12	12,12	11,11	10,10	14,14	6,6	X,X	11,11	11,13	-
0.93(26/28)	601	KYO-1	10,13	12,12	11,11	10,10	14,14	6,7	X,X	9,11	11,14	-
0.86(24/28)	266	S-117	11,11	12,12	11,11	11,14	14,14	6,6	X,X	8,8	13,13	-
0.86(24/28)	RCB2097	TE-4	10,10	11,11	11,11	9,10	14,14	6,6	X,X	8,11	11,11	-
0.79(22/28)	68	NAMALWA.KN2	12,13	12,12	11,11	9,9	14,14	7,9.3	X,X	6,11	10,11	-
0.79(22/28)	443	CAL-12T	12,12	11,11	10,11	10,10	16,16	6,6	X,X	8,8	13,13	-
0.79(22/28)	661	UPCI-SCC-172	12,12	11,11	9,11	10,10	17,18	9.3,9.3	X,X	11,11	11,11	-
0.79(22/28)	674	LAN-6	10,12	12,12	11,12	10,12	14,16	6,6	X,X	8,8	11,12	-
0.79(22/28)	721	NCEB-1	9,11	12,12	10,11	9,10	14,18	6,9.3	X,X	11,11	12,13	-
0.79(22/28)	CRL-2293	SJO	10,12	8,12	11,11	9,11	14,18	7,7	X,X	11,11	11,13	-

Figure 4. Authentication of the species of the sample

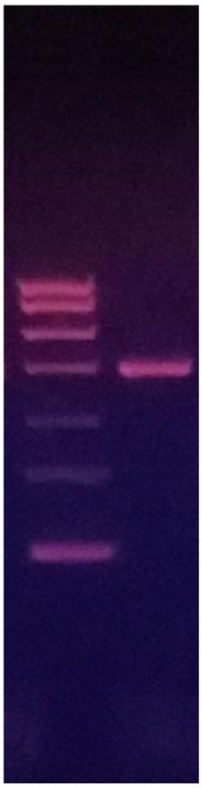

M: Marker. As the size of 700, 600, 500, 400, 300, 200 and 100bp from up to down.

Nine species are checked, as follow: *Homo sapiens* 391bp, *Cricetulus griseus*315bp, *Macaca mulatta*287bp, *Cercopithecus aethiops*222bp, *Rattus norvegicus*196bp, *Canis familiaris*172bp, *Mus musculus*150bp, *Bos Taurus*102bp, IC 70bp

The sample: The band size is 391bp which matches the size of human.

Report of Human Cell Line Authentication

（Notice:This authentication report is restricted to the cell sold from Guangzhou Cellcook Biotech Co., Ltd, and the date with seal is the date of delivery. ）

Ⅰ. Sample

Sample Name: labeled as 'ZR-75-1'

Ⅱ. Method and Procedure

1. PCR is amplified with STR Multi-amplification Kit (PowerPlex™16HS System);
2. PCR products are assayed with 3100 DNA Analyzer (Applied Biosystems®).
3. Amplification of gene COX1 and electrophoresis are employed to survey the species of the sample.

Ⅲ. Results

1. The STR profiles of the cell line sample are in the attached table and figure.
2. The search result in ATCC and DSMZ databases.
3. The electrophoresis figure of gene COX1.

ZR-75-1: ①No loci has tri-alleles or tetra-alleles. Contamination of other human cell line is not found (Figure 1 & Table 1). ②100% matched cell lines are found in ATCC and DSMZ data banks. And the cell line named as "ZR-75-1" *et al*. (Figure 2 & Figure 3). ③The sample is a human cell line. Contamination of other species cells are not found in the sample (Figure 4).

Operator: Xiaohua Mo

Auditor: Xuanyi Liang

Guangzhou Cellcook Biotech Co., Ltd

GUANGZHOU CELLCOOK BIOTECH CO.,LTD
12 JUN 2018
CELL STR PROFILING
PASS

Figure 1. STR profiles of ZR-75-1 cell line

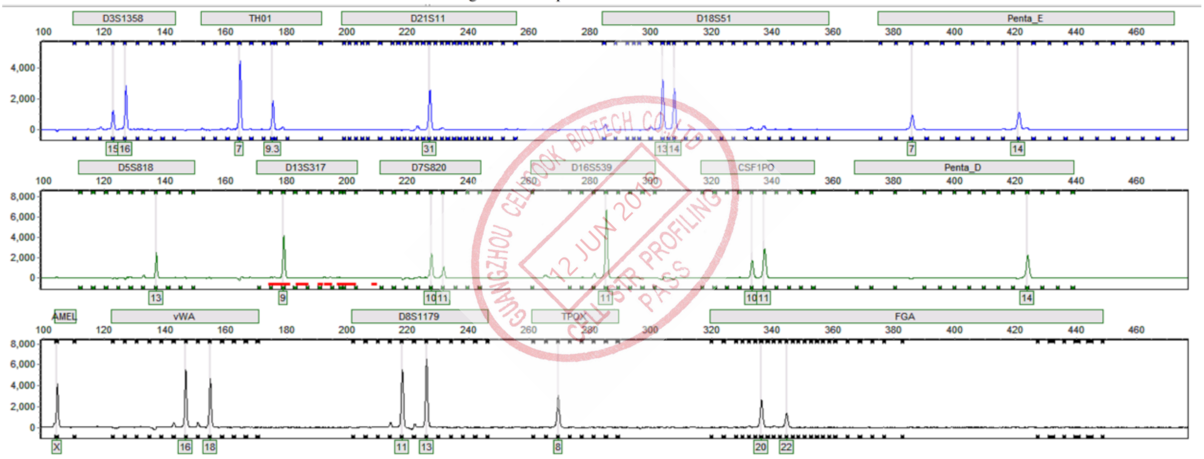

Table 1. STR profiles of ZR-75-1 cell line

	Allele1	Allele2
D3S1358	15	16
TH01	7	9.3
D21S11	31	
D18S51	13	14
Penta_E	7	14
D5S818	13	
D13S317	9	
D7S820	10	11
D16S539	11	
CSF1PO	10	11
Penta_D	14	
AMEL	x	
vWA	16	18
D8S1179	11	13
TPOX	8	
FGA	20	22

GUANGZHOU CELLCOOK BIOTECH CO.,LTD
12 JUN 2018
CELL STR PROFILING
PASS

Figure 2. Search result in ATCC database

SEARCH THE STR DATABASE

As part of our continuing efforts to characterize and authenticate the cell lines in the Cell Biology collection, ATCC has developed a comprehensive database of short tandem repeat (STR) DNA profiles for all of our human cell lines. View our brief tutorial before starting.

1. STR Profiling Analysis
2. Matching Algorithm
3. Interrogating the Database

Showing 1 - 1 Of 1

PageSize: 100 ▼

Add to Cart	%Match	ATCC® Number	Designation	D5S818	D13S317	D7S820	D16S539	vWA	TH01	AMEL	TPOX	CSF1PO
☐	100.0	CRL-1500	ZR-75-1Breast CarcinomaHuman	13	9	10,11	11	16,18	7,9.3	X	8	10,11

Figure 3. Search result in DSMZ database

Result of STR matching analysis by your data.

- DSMZ Profile Database -

A graphical presentation is shown at the bottom of this page.

EV	Cell No.	Cell name	Locus names									Figures
			D5S818	D13S317	D7S820	D16S539	VWA	TH01	AM	TPOX	CSF1PO	
	Query (Your Cell)		13	9	10,11	11	16,18	7,9.3	x	8	10,11	
1.16(36/31)	CRL-1500	ZR-75-1	13,13	9,9	10,11	11,11	16,18	7,9.3	X,X	8,8	10,11	-
1.16(36/31)	JCRB0823	YMB-1	13,13	9,9	10,11	11,11	16,18	7,9.3	X,X	8,8	10,11	-
1.16(36/31)	JCRB0825	YMB-1-E	13,13	9,9	10,11	11,11	16,18	7,9.3	X,X	8,8	10,11	-
1.16(36/31)	RCB1906	ZR-75-1	13,13	9,9	10,11	11,11	16,18	7,9.3	X,X	8,8	10,11	-
0.84(26/31)	450	BC-3C	11,13	9,9	11,11	11,13	15,16	6,9	X,X	8,8	11,11	-
0.84(26/31)	494	HDQ-P1	11,11	12,12	11,12	11,11	16,18	9.3,9.3	X,X	8,8	11,11	-
0.84(26/31)	CRL-10782	MCF-12A	11,13	9,11	8,11	9,12	18,18	7,7	X,X	8,8	10,11	-
0.84(26/31)	CRL-10783	MCF-12F	11,13	9,11	8,11	9,12	18,18	7,7	X,X	8,8	10,11	-
0.84(26/31)	CRL-2020	DBTRG-05MG	12,13	9,9	11,11	10,12	15,16	7,8	X,X	8,8	10,11	-
0.84(26/31)	CRL-2090	CCD-1074Sk	10,11	9,9	10,11	11,11	16,17	9,9.3	X,X	8,8	11,12	-
0.84(26/31)	CRL-5934	NCI-H2227	13,13	8,8	10,12	9,9	16,18	9.3,9.3	X,X	8,8	11,11	-

Figure 4. Authentication of the species of the sample

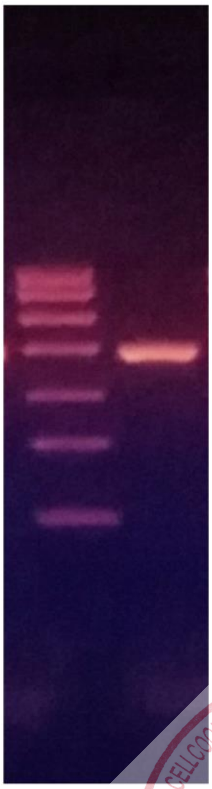

M: Marker. As the size of 700, 600, 500, 400, 300, 200 and 100bp from up to down.

Nine species are checked, as follow: *Homo sapiens* 391bp, *Cricetulus griseus* 315bp, *Macaca mulatta* 287bp, *Cercopithecus aethiops* 222bp, *Rattus norvegicus* 196bp, *Canis familiaris* 172bp, *Mus musculus* 150bp, *Bos Taurus* 102bp, IC 70bp

The sample: The band size is 391bp which matches the size of human.

Report of Human Cell Line Authentication

Ⅰ. Sample

Sample Name: labeled as ‘MDA-MB-453’

Ⅱ. Method and Procedure

1. PCR is amplified with STR Multi-amplification Kit (PowerPlex™16HS System);
2. PCR products are assayed with 3100 DNA Analyzer (Applied Biosystems®).
3. Amplification of gene COX1 and electrophoresis are employed to survey the species of the sample.

Ⅲ. Results

1. The STR profiles of the cell line sample are in the attached table and figure.
2. The search result in ATCC and DSMZ databases.
3. The electrophoresis figure of gene COX1.

MDA-MB-453: ①No loci has tri-alleles or tetra-alleles. Contamination of other human cell lines are not found (Figure 1 & Table 1). ②Compared the STR data of MDA-MB-453 cell line in the databases of ATCC and DSMZ, all the alleles of MDA-MB-453 were exactly matched with the alleles of MDA-MB-453 cells found in both cell banks (Figure 2&3). ③The sample is a human cell line. Contamination of other species cells are not found in the sample (Figure 4).

To all above, the sample is a single cell line, and it is MDA-MB-453 cell line.

Operator: Xiaohua Mo Auditor: Xuanyi Liang

Guangzhou Cellcook Biotech Co., Ltd

GUANGZHOU CELLCOOK BIOTECH CO.,LTD
15 APR 2019
CELL STR PROFILING
PASS

（Notice:This authentication report is restricted to the cell sold from Guangzhou Cellcook Biotech Co., Ltd, and the date with seal is the date of delivery.）

Figure 1. STR profiles of MDA-MB-453 cell line

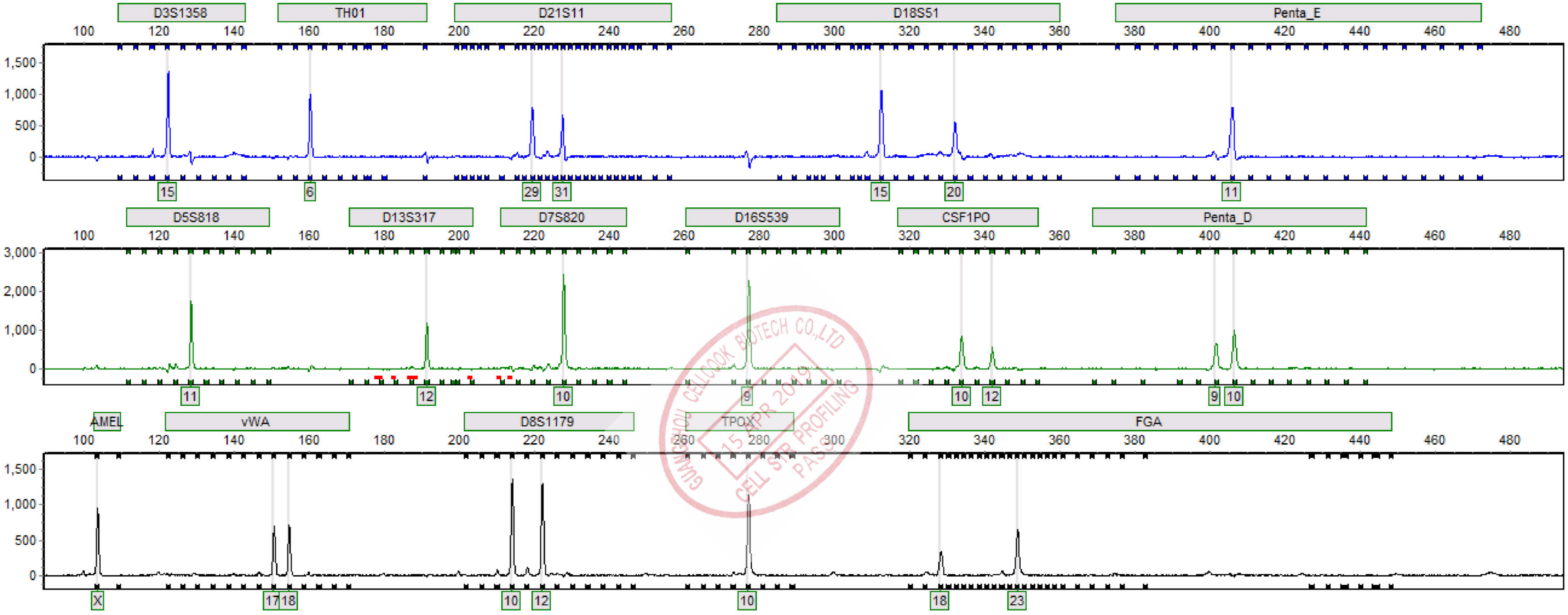

Table 1. STR profiles of MDA-MB-453 cell line

21	Allele1	Allele2
D3S1358	15	
TH01	6	
D21S11	29	31
D18S51	15	20
Penta_E	11	
D5S818	11	
D13S317	12	
D7S820	10	
D16S539	9	
CSF1PO	10	12
Penta_D	9	10
AMEL	x	
vWA	17	18
D8S1179	10	12
TPOX	10	
FGA	18	23

Figure 2. Search result in ATCC database

SEARCH THE STR DATABASE

As part of our continuing efforts to characterize and authenticate the cell lines in the Cell Biology collection, ATCC has developed a comprehensive database of short tandem repeat (STR) DNA profiles for all of our human cell lines. View our brief tutorial before starting.

1. STR Profiling Analysis
2. Matching Algorithm
3. Interrogating the Database

Showing 1 - 2 Of 2

PageSize: 100

Add to Cart	%Match	ATCC® Number	Designation	D5S818	D13S317	D7S820	D16S539	vWA	TH01	AMEL	TPOX	CSF1PO
☐	100.0	HTB-131	MDA-MB-453Breast CarcinomaHuman	11	12	10	9	17,18	6	X	10	10,12
☐	100.0	CRL-2713	MDA-kb2Breast CarcinomaHuman	11	12	10	9	17,18	6	X	10	10,12

Add to Cart Export to Excel

Disclaimer: Reference to this database and the data contained therein may be cited in publications, and ATCC encourages such citation or reference. While every reasonable effort has been made to assure the accuracy of these data, no warranty, express or implied, is made by ATCC as to their accuracy.

Figure 3. Search result in DSMZ database

Result of STR matching analysis by your data.

- DSMZ Profile Database -

A graphical presentation is shown at the bottom of this page.

EV	Cell No.	Cell name	Locus names									Figures
			D5S818	D13S317	D7S820	D16S539	VWA	TH01	AM	TPOX	CSF1PO	
	Query (Your Cell)		*11,11*	*12,12*	*10,10*	*9,9*	*17,18*	*6,6*	*x,x*	*10,10*	*10,12*	
1.00(36/36)	65	MDA-MB-453	11,11	12,12	10,10	9,9	17,18	6,6	X,X	10,10	10,12	-
1.00(36/36)	CRL-2713	MDA-kb2	11,11	12,12	10,10	9,9	17,18	6,6	X,X	10,10	10,12	-
1.00(36/36)	HTB-131	MDA-MB-453	11,11	12,12	10,10	9,9	17,18	6,6	X,X	10,10	10,12	-
1.00(36/36)	RCB1192	MDA-MB-453	11,11	12,12	10,10	9,9	17,18	6,6	X,X	10,10	10,12	-
0.70(26/37)	529	OCI-M1	11,11	11,12	10,10	9,9	17,18	9,9	X,X	11,11	10,12,14	-
0.67(24/36)	HTB-4	T24	10,12	12,12	10,11	9,9	17,17	6,6	X,X	8,11	10,12	-
0.67(24/36)	JCRB0133	KHM-1B	11,11	11,11	10,10	9,9	14,17	6,6	X,X	11,11	10,10	-
0.67(24/36)	JCRB0710	EJ-1	10,12	12,12	10,11	9,9	17,17	6,6	X,X	8,11	10,12	-
0.67(24/36)	JCRB0711	T24	10,12	12,12	10,11	9,9	17,17	6,6	X,X	8,11	10,12	-

Figure 4. Authentication of the species of the sample

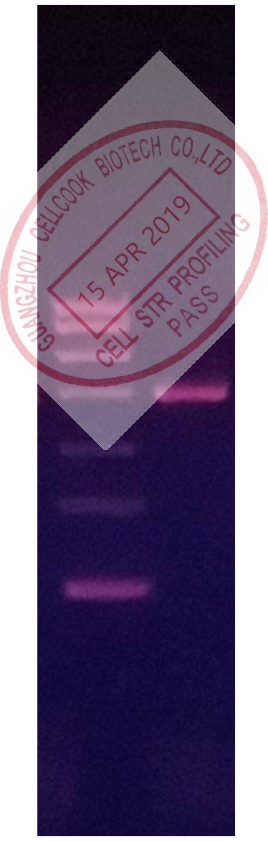

M: Marker. As the size of 700, 600, 500, 400, 300, 200 and 100bp from up to down.

Nine species are checked, as follow: *Homo sapiens* 391bp, *Cricetulus griseus*315bp, *Macaca mulatta*287bp, *Cercopithecus aethiops*222bp, *Rattus norvegicus*196bp, *Canis familiaris*172bp, *Mus musculus*150bp, *Bos Taurus*102bp, IC 70bp

The sample: The band size is 391bp which matches the size of human.

Report of Human Cell Line Authentication

I. Sample

Sample Name: labeled as 'HCC38'

II. Method and Procedure

1. PCR is amplified with STR Multi-amplification Kit (PowerPlex™16HS System);
2. PCR products are assayed with 3100 DNA Analyzer (Applied Biosystems®).
3. Amplification of gene COX1 and electrophoresis are employed to survey the species of the sample.

III. Results

1. The STR profiles of the cell line sample are in the attached table and figure.
2. The search result in ATCC and DSMZ databases.
3. The electrophoresis figure of gene COX1.

HCC38: ①No loci has tri-alleles or tetra-alleles. Contamination of other human cell lines are not found (Figure 1 & Table 1). ②Compared the STR data of HCC38 cell line in the databases of ATCC and DSMZ, all the alleles of HCC38 were exactly matched with the alleles of HCC38 cells found in both cell banks (Figure 2&3). ③The sample is a human cell line. Contamination of other species cells are not found in the sample (Figure 4).

To all above, the sample is a single cell line, and it is HCC38 cell line.

Operator: Xiaohua Mo   Auditor: Xuanyi Liang

Guangzhou Cellcook Biotech Co., Ltd

GUANGZHOU CELLCOOK BIOTECH CO.,LTD
28 APR 2019
CELL STR PROFILING
PASS

（Notice:This authentication report is restricted to the cell sold from Guangzhou Cellcook Biotech Co., Ltd, and the date with seal is the date of delivery. ）

Figure 1. STR profiles of HCC38 cell line

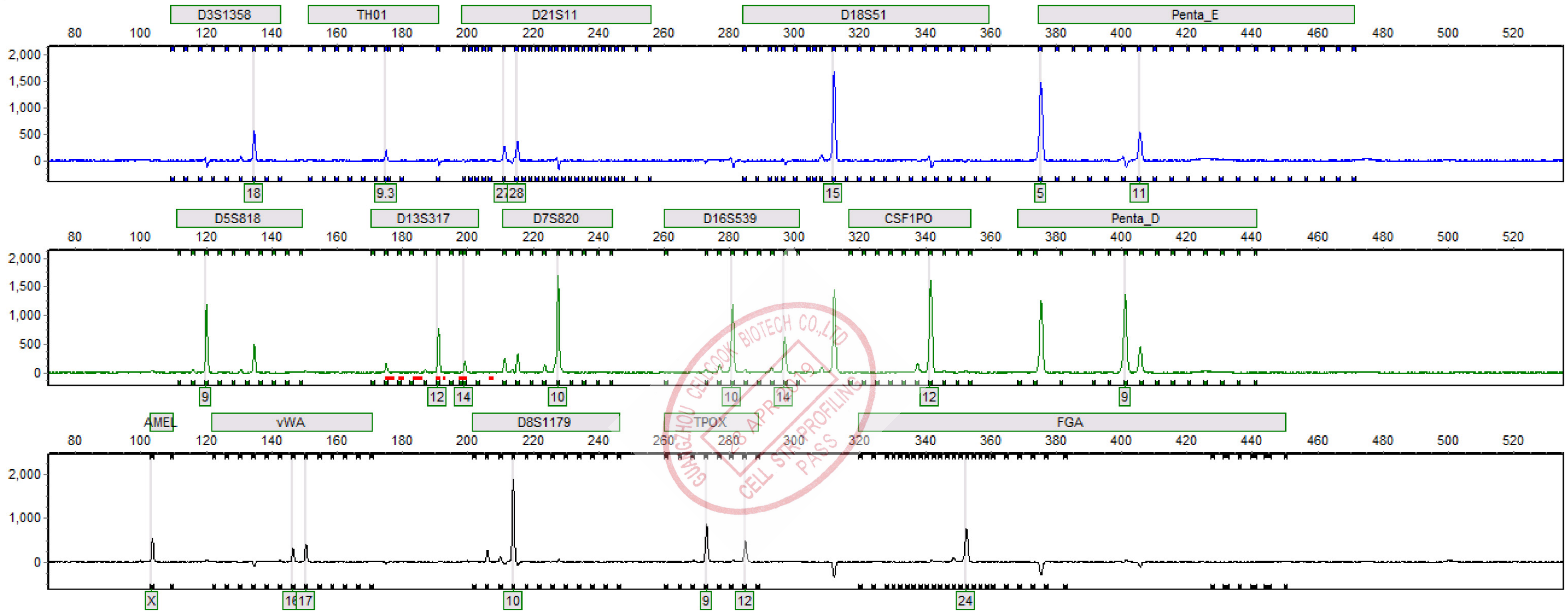

Table 1. STR profiles of HCC38 cell line

	Allele1	Allele2
D3S1358	18	
TH01	9.3	
D21S11	27	28
D18S51	15	
Penta_E	5	11
D5S818	9	
D13S317	12	14
D7S820	10	
D16S539	10	14
CSF1PO	12	
Penta_D	9	
AMEL	x	
vWA	16	17
D8S1179	10	
TPOX	9	12
FGA	24	

Figure 2. Search result in ATCC database

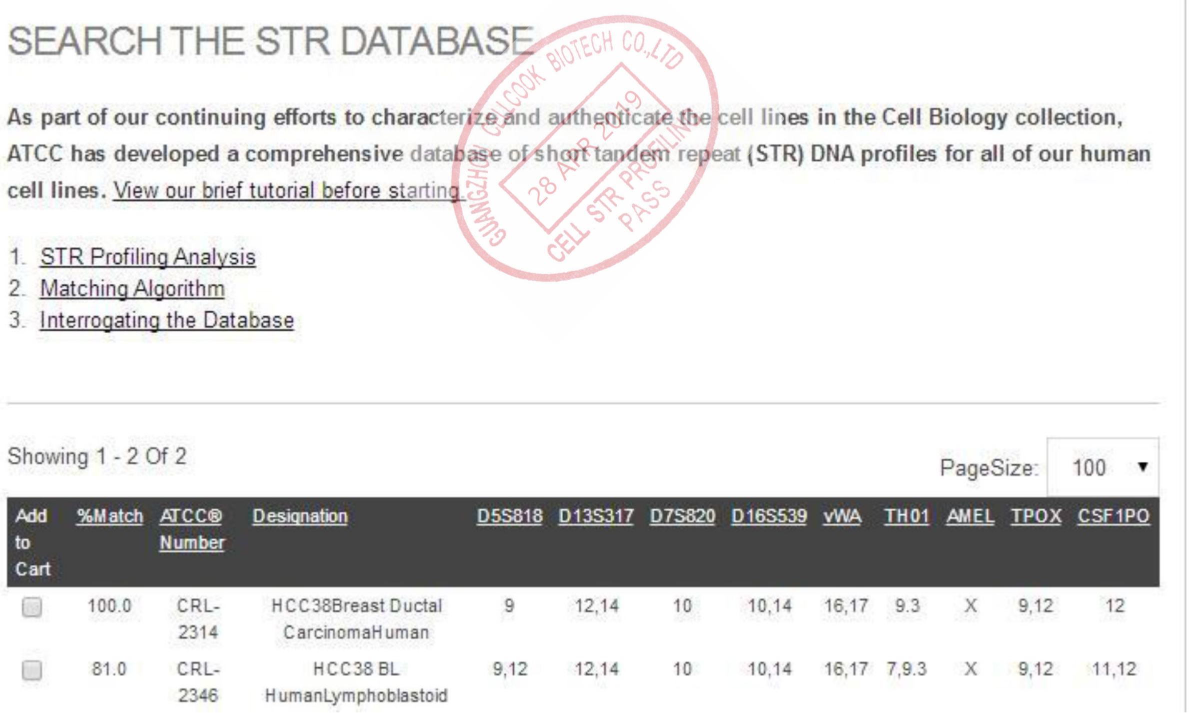

SEARCH THE STR DATABASE

As part of our continuing efforts to characterize and authenticate the cell lines in the Cell Biology collection, ATCC has developed a comprehensive database of short tandem repeat (STR) DNA profiles for all of our human cell lines. View our brief tutorial before starting.

1. STR Profiling Analysis
2. Matching Algorithm
3. Interrogating the Database

Showing 1 - 2 Of 2

PageSize: 100

Add to Cart	%Match	ATCC® Number	Designation	D5S818	D13S317	D7S820	D16S539	vWA	TH01	AMEL	TPOX	CSF1PO
	100.0	CRL-2314	HCC38Breast Ductal CarcinomaHuman	9	12,14	10	10,14	16,17	9.3	X	9,12	12
	81.0	CRL-2346	HCC38 BL HumanLymphoblastoid	9,12	12,14	10	10,14	16,17	7,9.3	X	9,12	11,12

GUANGZHOU CELLCOOK BIOTECH CO.,LTD
28 APR 2019
CELL STR PROFILING
PASS

Figure 3. Search result in DSMZ database

Result of STR matching analysis by your data.

- DSMZ Profile Database -

A graphical presentation is shown at the bottom of this page.

EV	Cell No.	Cell name	Locus names									Figures
			D5S818	D13S317	D7S820	D16S539	VWA	TH01	AM	TPOX	CSF1PO	
	Query (Your Cell)		*9,9*	*12,14*	*10,10*	*10,14*	*16,17*	*9.3,9.3*	*x,x*	*9,12*	*12,12*	
1.00(36/36)	CRL-2314	HCC38	9,9	12,14	10,10	10,14	16,17	9.3,9.3	X,X	9,12	12,12	-
0.83(30/36)	CRL-2346	HCC 38BL	9,12	12,14	10,10	10,14	16,17	7,9.3	X,X	9,12	11,12	-

Figure 4. Authentication of the species of the sample

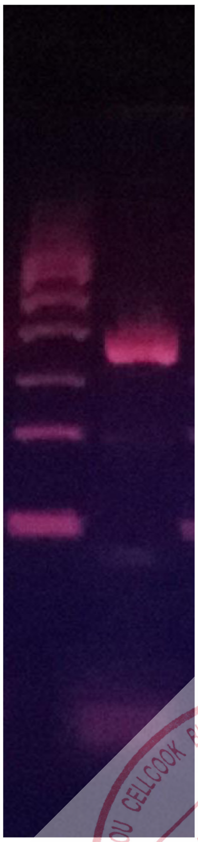

GUANGZHOU CELLCOOK BIOTECH CO.,LTD 28 APR 2019 CELL STR PROFILING PASS

M: Marker. As the size of 700, 600, 500, 400, 300, 200 and 100bp from up to down.

Nine species are checked, as follow: *Homo sapiens* 391bp, *Cricetulus griseus*315bp, *Macaca mulatta*287bp, *Cercopithecus aethiops*222bp, *Rattus norvegicus*196bp, *Canis familiaris*172bp, *Mus musculus*150bp, *Bos Taurus*102bp, IC 70bp

The sample: The band size is 391bp which matches the size of human.

Report of Human Cell Line Authentication

I. Sample

Sample Name: labeled as ‘BT-20’

II. Method and Procedure

1. PCR is amplified with STR Multi-amplification Kit (PowerPlex 21 System);
2. PCR products are assayed with 3100 DNA Analyzer (Applied Biosystems®).
3. Amplification of gene COX1 and electrophoresis are employed to survey the species of the sample.

III. Results

1. The STR profiles of the cell line sample are in the attached table and figure.
2. The search result in ATCC and DSMZ databases.
3. The electrophoresis figure of gene COX1.

BT-20: ①No loci has tri-alleles or tetra-alleles. Contamination of other human cell lines are not found (Figure 1 & Table 1). ②Compared the STR data of BT-20 cell line in the databases of ATCC and DSMZ, all the alleles of BT-20 were exactly matched with the alleles of BT-20 cells found in both cell banks (Figure 2&3). ③The sample is a human cell line. Contamination of other species cells are not found in the sample (Figure 4).

To all above, the sample is a single cell line, and it is BT-20 cell line.

Operator: Xiaohua Mo Auditor: Xuanyi Liang

Guangzhou Cellcook Biotech Co., Ltd

GUANGZHOU CELLCOOK BIOTECH CO., LTD
23 JUL 2018
CELL STR PROFILING

（Notice:This authentication report is restricted to the cell sold from Guangzhou Cellcook Biotech Co., Ltd, and the date with seal is the date of delivery.）

Figure 1. STR profiles of BT-20cell line

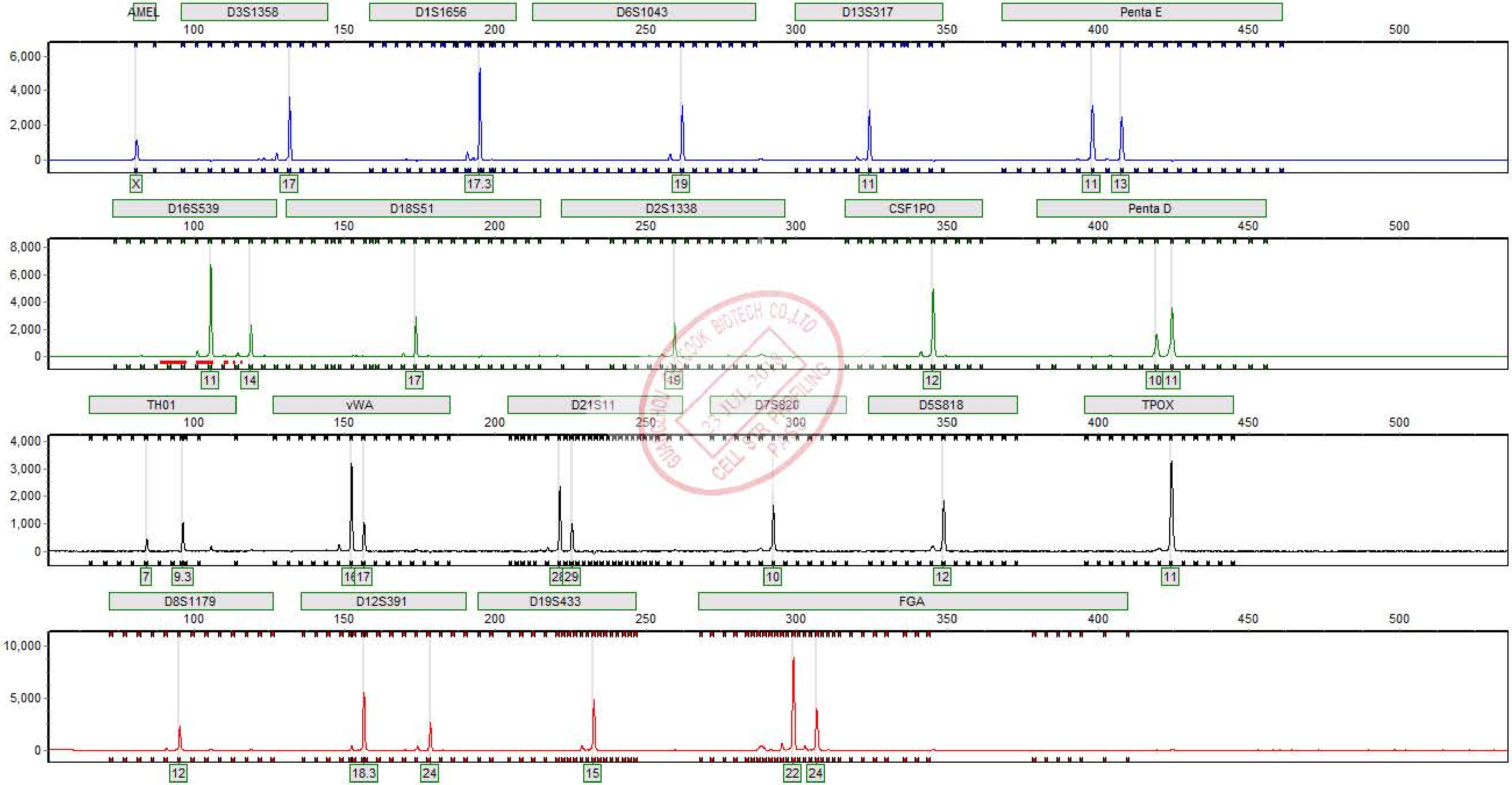

Table 1. STR profiles of BT-20 cell line

	Allele1	Allele2
AMEL	X	
D3S1358	17	
D1S1656	17.3	
D6S1043	19	
D13S317	11	
Penta E	11	13
D16S539	11	14
D18S51	17	
D2S1338	19	
CSF1PO	12	
Penta D	10	11
TH01	7	9.3
vWA	16	17
D21S11	28	29
D7S820	10	
D5S818	12	
TPOX	11	
D8S1179	12	
D12S391	18.3	24
D19S433	15	
FGA	22	24

Figure 2. Search result in ATCC database

SEARCH THE STR DATABASE

As part of our continuing efforts to characterize and authenticate the cell lines in the Cell Biology collection, ATCC has developed a comprehensive database of short tandem repeat (STR) DNA profiles for all of our human cell lines. View our brief tutorial before starting.

1. STR Profiling Analysis
2. Matching Algorithm
3. Interrogating the Database

Showing 1 - 1 Of 1

PageSize: 100

Add to Cart	%Match	ATCC® Number	Designation	D5S818	D13S317	D7S820	D16S539	vWA	TH01	AMEL	TPOX	CSF1PO
	100.0	HTB-19	BT-20Breast CarcinomaHuman	12	11	10	11,14	16,17	7,9.3	X	11	12

Figure 3. Search result in DSMZ database

Result of STR matching analysis by your data.

- DSMZ Profile Database -

A graphical presentation is shown at the bottom of this page.

EV	Cell No.	Cell name	Locus names									Figures
			D5S818	D13S317	D7S820	D16S539	VWA	TH01	AM	TPOX	CSF1PO	
	Query (Your Cell)		*12,12*	*11,11*	*10,10*	*11,14*	*16,17*	*7,9.3*	*x,x*	*11,11*	*12,12*	
1.00(36/36)	HTB-19	BT-20	12,12	11,11	10,10	11,14	16,17	7,9.3	X,X	11,11	12,12	-

Figure 4. Authentication of the species of the sample

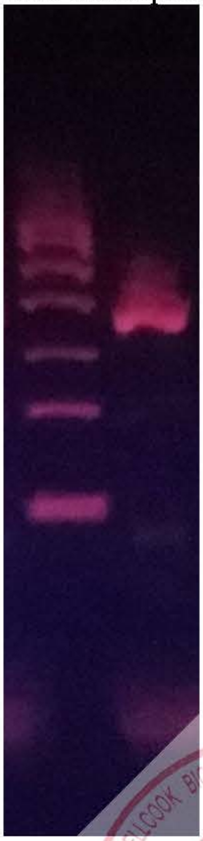

M: Marker. As the size of 700, 600, 500, 400, 300, 200 and 100bp from up to down.

Nine species are checked, as follow: *Homo sapiens* 391bp, *Cricetulus griseus*315bp, *Macaca mulatta*287bp, *Cercopithecus aethiops*222bp, *Rattus norvegicus*196bp, *Canis familiaris*172bp, *Mus musculus*150bp, *Bos Taurus*102bp, IC 70bp

The sample: The band size is 391bp which matches the size of human.

Report of Human Cell Line Authentication

（Notice:This authentication report is restricted to the cell sold from Guangzhou Cellcook Biotech Co., Ltd, and the date with seal is the date of delivery. ）

Ⅰ. Sample

Sample Name: labeled as ‘MDA-MB-231’

Ⅱ. Method and Procedure

1. PCR is amplified with STR Multi-amplification Kit (PowerPlex^{TM}16HS System);
2. PCR products are assayed with 3100 DNA Analyzer (Applied Biosystems®).
3. Amplification of gene COX1 and electrophoresis are employed to survey the species of the sample.

Ⅲ. Results

1. The STR profiles of the cell line sample are in the attached table and figure.
2. The search result in ATCC and DSMZ databases.
3. The electrophoresis figure of gene COX1.

MDA-MB-231: ①No loci has tri-alleles or tetra-alleles. Contamination of other human cell line is not found (Figure 1 & Table 1). ②100% matched cell lines are found in ATCC and DSMZ data banks. And the cell line named as “MDA-MB-231” *et al*. (Figure 2 & Figure 3). ③ The sample is a human cell line. Contamination of other species cells are not found in the sample (Figure 4).

Operator: Xiaohua Mo

Auditor: Xuanyi Liang

Guangzhou Cellcook Biotech Co., Ltd

GUANGZHOU CELLCOOK BIOTECH CO.,LTD
12 JUN 2018
CELL STR PROFILING
PASS

Figure 1. STR profiles of MDA-MB-231 cell line

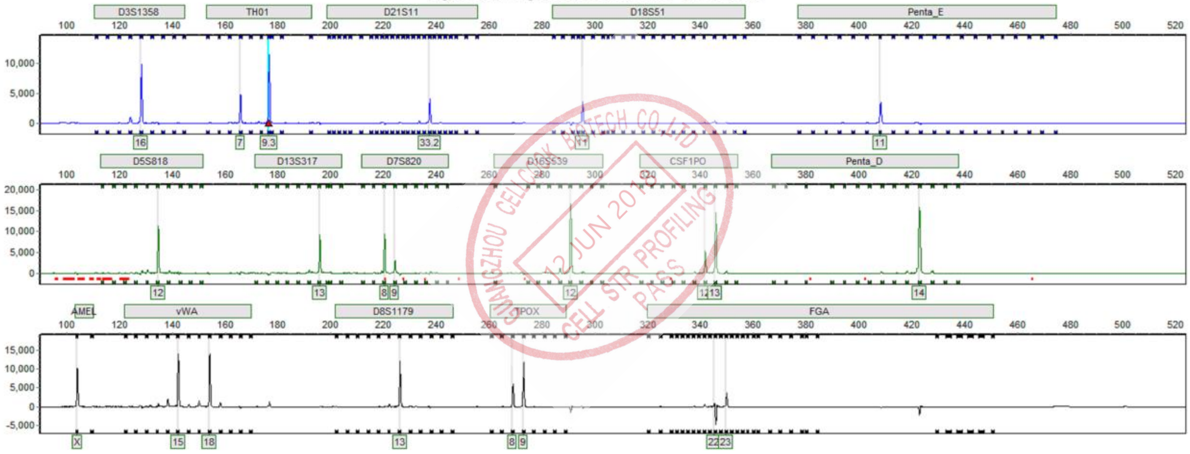

Table 1. STR profiles of MDA-MB-231 cell line

	Allele1	Allele2
D3S1358	16	17
TH01	7	9.3
D21S11	33.2	
D18S51	11	
Penta_E	11	
D5S818	12	
D13S317	13	
D7S820	8	9
D16S539	12	
CSF1PO	12	13
Penta_D	14	
AMEL	x	
vWA	15	18
D8S1179	13	
TPOX	8	9
FGA	22	23

GUANGZHOU CELLCOOK BIOTECH CO.,LTD
12 JUL 2018
CELL STR PROFILING PASS

Figure 2. Search result in ATCC database

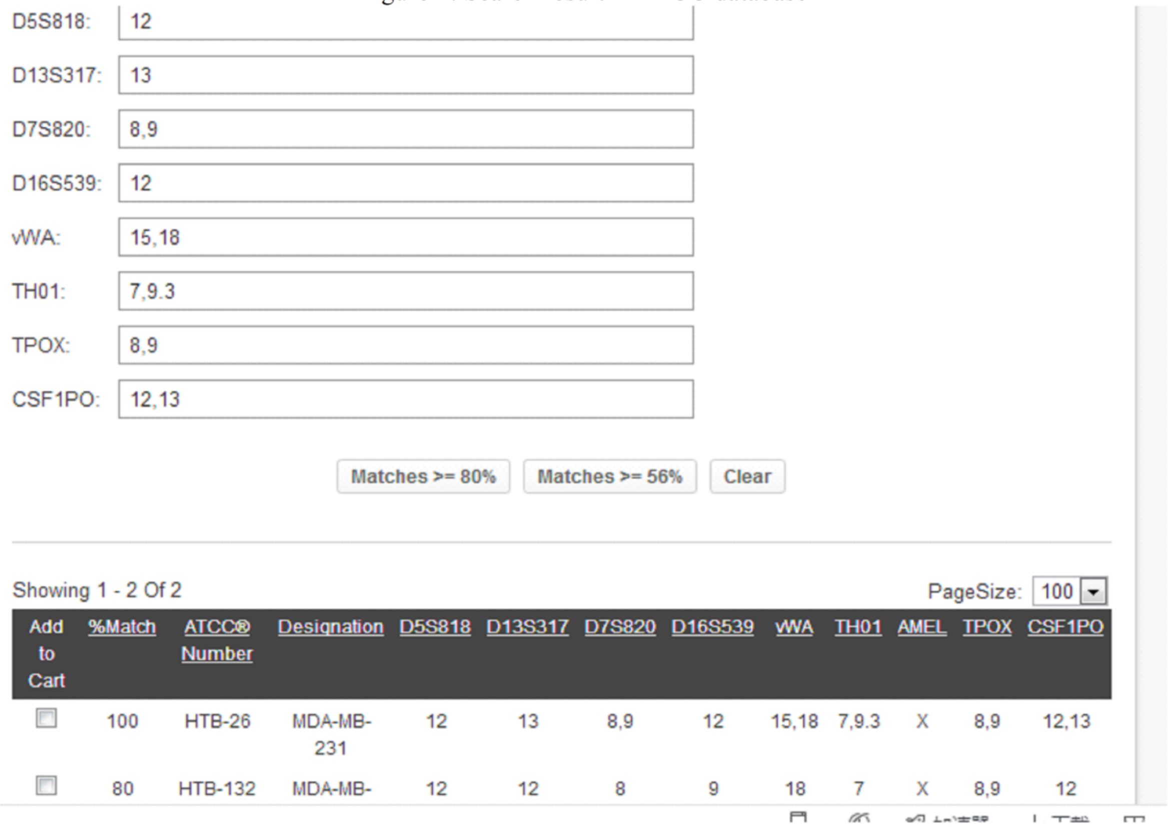

Figure 3. Search result in DSMZ database

Result of STR matching analysis by your data.
- DSMZ Profile Database -

A graphical presentation is shown at the bottom of this page.

EV	Cell No.	Cell name	Locus names									Figures
			D5S818	D13S317	D7S820	D16S539	VWA	TH01	AM	TPOX	CSF1PO	
	Query (Your Cell)		12	13	8, 9	12	15, 18	7, 9.3	x	8, 9	12, 13	
1.12(36/32)	HTB-26	MDA-MB-231	12,12	13,13	8,9	12,12	15,18	7,9.3	X,X	8,9	12,13	-
1.12(36/32)	732	MDA-MB-231	12,12	13,13	8,9	12,12	15,18	7,9.3	X,X	8,9	12,13	-
0.88(28/32)	383	EPLC-272H	13,13	12,13	8,9	11,12	18,18	7,9.3	X,X	8,8	13,13	-
0.88(28/32)	CCL-235	SW837 [SW-837]	12,12	13,13	9,12	12,12	15,16	9.3,9.3	X,X	8,9	10,10	-
0.88(28/32)	CRL-1593.2	U-937	12,12	10,12	9,11	12,12	15,15	9.3,9.3	X,X	8,11	12,12	-
0.88(28/32)	HTB-132	MDA-MB-468	12,12	12,12	8,8	9,9	18,18	7,7	X,X	8,9	12,12	-
0.88(28/32)	738	MDA-MB-468	12,12	12,12	8,8	9,9	18,18	7,7	X,X	8,9	12,12	-
0.81(26/32)	CRL-10296	NCI-H295 [H295]	12,12	13,13	9,12	11,11	17,18	9.3,9.3	X,X	8,8	10,12	-
0.81(26/32)	CRL-2128	NCI-H295R [H295R]	12,12	13,13	9,12	11,11	17,18	9.3,9.3	X,X	8,8	10,12	-
0.81(26/32)	CRL-5908	NCI-H1975 [H-1975, H1975]	11,12	10,13	8,11	9,12	18,18	7,7	X,X	8,11	12,12	-
0.81(26/32)	CRL-5918	NCI-H2073 [H2073]	12,12	13,13	12,12	12,12	17,17	6,9.3	X,X	9,9	13,13	-

Figure 4. Authentication of the species of the sample

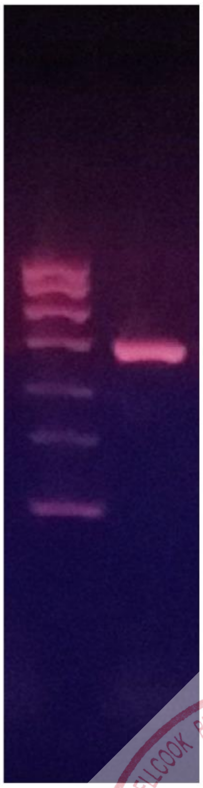

CELLCOOK BIOTECH CO.,LTD
12 JUN 2018
CELL STR PROFILING

M: Marker. As the size of 700, 600, 500, 400, 300, 200 and 100bp from up to down.

Nine species are checked, as follow: *Homo sapiens* 391bp, *Cricetulus griseus*315bp, *Macaca mulatta*287bp, *Cercopithecus aethiops*222bp, *Rattus norvegicus*196bp, *Canis familiaris*172bp, *Mus musculus*150bp, *Bos Taurus*102bp, IC 70bp

The sample: The band size is 391bp which matches the size of human.

Report of Human Cell Line Authentication

（Notice:This authentication report is restricted to the cell sold from Guangzhou Cellcook Biotech Co., Ltd, and the date with seal is the date of delivery. ）

Ⅰ. Sample

Sample Name: labeled as ‘BT-549’

Ⅱ. Method and Procedure

1. PCR is amplified with STR Multi-amplification Kit (PowerPlex™16HS System);
2. PCR products are assayed with 3100 DNA Analyzer (Applied Biosystems®).
3. Amplification of gene COX1 and electrophoresis are employed to survey the species of the sample.

Ⅲ. Results

1. The STR profiles of the cell line sample are in the attached table and figure.
2. The search result in ATCC and DSMZ databases.
3. The electrophoresis figure of gene COX1.

BT-549: ①No loci has tri-alleles or tetra-alleles. Contamination of other human cell line is not found (Figure 1 & Table 1). ②100% matched cell lines are found in ATCC and DSMZ data banks. And the cell line named as “BT-579” *et al.* (Figure 2 & Figure 3). ③The sample is a human cell line. Contamination of other species cells are not found in the sample (Figure 4).

Operator: Xiaohua Mo　　　　Auditor: Xuanyi Liang

GUANGZHOU CELLCOOK BIOTECH CO.,LTD
09 OCT 2018
CELL STR PROFILING
PASS

Guangzhou Cellcook Biotech Co., Ltd

Figure 1. STR profiles of BT-549 cell line

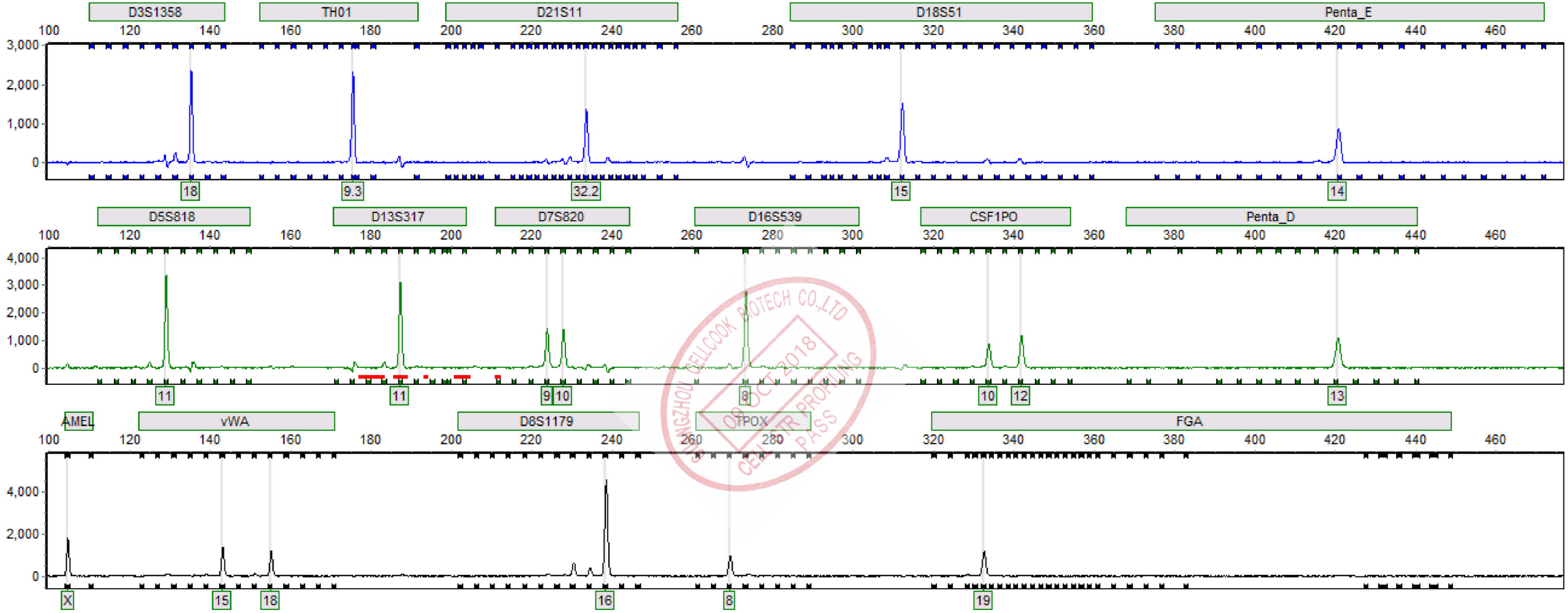

Table 1. STR profiles of BT-549 cell line

	Allele1	Allele2
D3S1358	16	
TH01	8	9.3
D21S11	29	
D18S51	14	17
Penta_E	7	11
D5S818	11	
D13S317	11	
D7S820	8	11
D16S539	11	12
CSF1PO	10	12
Penta_D	9	
AMEL	x	y
vWA	14	
D8S1179	13	14
TPOX	8	11
FGA	23	

BIOTECH CO.,LTD
09 OCT 2018
STR PROFILING
PASS

Figure 2. Search result in ATCC database

SEARCH THE STR DATABASE

As part of our continuing efforts to characterize and authenticate the cell lines in the Cell Biology collection, ATCC has developed a comprehensive database of short tandem repeat (STR) DNA profiles for all of our human cell lines. View our brief tutorial before starting.

1. STR Profiling Analysis
2. Matching Algorithm
3. Interrogating the Database

Showing 1 - 1 Of 1

PageSize: 100

Add to Cart	%Match	ATCC® Number	Designation	D5S818	D13S317	D7S820	D16S539	vWA	TH01	AMEL	TPOX	CSF1PO
☐	100.0	HTB-122	BT-549Breast Ductal CarcinomaHuman	11	11	9,10	8	15	9.3	X	8	10,12

Add to Cart | Export to Excel

Figure 3. Search result in DSMZ database

Result of STR matching analysis by your data.

- DSMZ Profile Database -

A graphical presentation is shown at the bottom of this page.

EV	Cell No.	Cell name	Locus names									Figures
			D5S818	D13S317	D7S820	D16S539	VWA	TH01	AM	TPOX	CSF1PO	
	Query (Your Cell)		*11*	*11*	*9,10*	*8*	*15,18*	*9.3*	*x*	*8*	*10,12*	
1.20(36/30)	HTB-122	BT-549	11,11	11,11	9,10	8,8	15,15	9.3,9.3	X,X	8,8	10,12	-
0.93(28/30)	HTB-134	Hs 766T	11,11	8,11	10,10	9,12	18,18	6,9.3	X,X	8,8	12,12	-
0.87(26/30)	440	CAL-85-1	11,11	11,11	9,10	11,13	18,18	6,6	X,X	8,11	12,12	-
0.87(26/30)	744	JOPACA-1	11,11	9,12	9,10	10,10	15,18	9,9.3	X,X	8,8	10,10	-
0.87(26/30)	CRL-4033	hAMSC83telo	11,12	9,11	9,10	9,12	18,18	8,9.3	X,X	8,8	10,12	-
0.80(24/30)	120	NC-NC	11,11	12,13	9,9	12,13	14,15	7,9.3	X,X	8,8	10,12	-
0.80(24/30)	179	PA-TU-8902	11,12	11,12	8,10	9,11	18,18	9.3,9.3	X,X	8,8	10,11	-
0.80(24/30)	242	GAMG	10,11	11,11	9,10	11,13	14,19	6,9.3	X,X	8,8	10,10	-
0.80(24/30)	291	U-138-MG	11,11	9,11	9,9	12,13	18,18	6,6	X,Y	8,8	12,12	-
0.80(24/30)	671	LAN-2	11,12	11,12	8,10	9,10	16,18	9.3,9.3	X,X	8,8	10,12	-
0.80(24/30)	734	Calu-6	11,11	11,11	10,10	13,13	17,17	9,9	X,X	8,8	12,12	-
0.80(24/30)	CCL-90	Cri du Chat	10,11	11,11	8,11	11,12	15,16	9.3,9.3	X,X	8,8	10,12	-
0.80(24/30)	CRL-2807	WM35	11,12	11,11	10,13	12,13	17,17	9.3,9.3	X,X	8,8	10,10	-
[illegible]	[illegible]	[illegible]	[illegible]	[illegible]	[illegible]	[illegible]	[illegible]	[illegible]	[illegible]	[illegible]	[illegible]	[illegible]

Figure 4. Authentication of the species of the sample

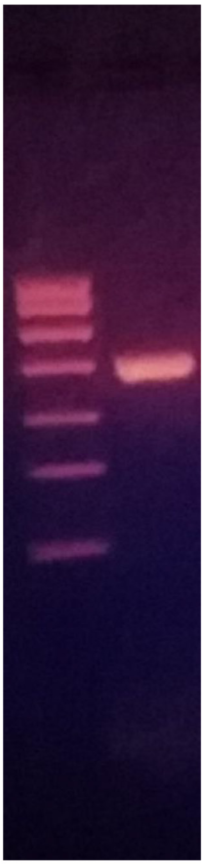

M: Marker. As the size of 700, 600, 500, 400, 300, 200 and 100bp from up to down.

Nine species are checked, as follow: *Homo sapiens* 391bp, *Cricetulus griseus*315bp, *Macaca mulatta*287bp, *Cercopithecus aethiops*222bp, *Rattus norvegicus*196bp, *Canis familiaris*172bp, *Mus musculus*150bp, *Bos Taurus*102bp, IC 70bp

The sample: The band size is 391bp which matches the size of human.
